# The osteoclast paradox in type 2 diabetes: low turnover, skeletal fragility, and implications for osteoclast-targeted therapy

**DOI:** 10.3389/fendo.2026.1972283

**Published:** 2026-10-30

**Authors:** Ziang Jia, Kuan Liang, Yali Dai

**Affiliations:** 1 Department of Cadre Health Care, Peking University Shenzhen Hospital Affiliated to Shenzhen University, Shenzhen, Guangdong, China; 2 Medical School, Shenzhen University, Shenzhen, Guangdong, China

**Keywords:** advanced glycation end products, antiresorptive therapy, bone remodeling, diabetic bone fragility, low bone turnover, osteoanabolic therapy, osteoclast, type 2 diabetes mellitus

## Abstract

Type 2 diabetes mellitus (T2DM) is increasingly recognized as a skeletal-fragility condition characterized at the population level by lower circulating markers of bone formation and resorption, frequently preserved or increased DXA-derived areal bone mineral density (aBMD), and increased fracture risk. These observations arise from different measurement levels and have not been demonstrated to constitute a single biological phenotype within the same individuals. In this Review, we use the term “osteoclast paradox” as an author-proposed heuristic framework—not a diagnostic entity or an established mechanistic syndrome—to organize apparent dissociations among circulating bone-turnover markers, osteoclast-lineage biology, tissue-level remodeling, matrix properties, and treatment response in T2DM. Direct human evidence linking these levels remains limited. A small exploratory human study suggests a possible dissociation between osteoclast precursor phenotype and mature function, whereas histomorphometry indicates that remodeling can remain coupled despite delayed progression; neither observation establishes generalized preservation or enhancement of mature osteoclast resorptive function. Experimental studies further show context-dependent effects of hyperglycemia, advanced glycation end products, oxidative stress, insulin signaling, inflammation, and the marrow environment on osteoclast differentiation and function. The observed skeletal phenotype is also modified by obesity and body composition, renal and microvascular disease, and glucose-lowering or weight-loss therapies; these overlapping exposures should not be attributed automatically to T2DM itself. Matrix glycation, altered material behavior, osteocyte dysfunction, osteoblast-lineage impairment, and altered basic multicellular unit kinetics may contribute to skeletal fragility despite low circulating turnover markers. Pooled randomized-trial analyses suggest that antiresorptive treatment responses are not obviously attenuated by T2DM, although diabetes-specific fracture estimates are unevenly powered and treatment response should not be interpreted as mechanistic validation of the proposed framework. Osteoanabolic studies demonstrate aBMD, trabecular bone score, and early microarchitectural responses, but diabetes-specific fracture endpoints remain limited. The framework is therefore intended to separate non-equivalent biological and clinical endpoints, identify evidence gaps, and generate testable hypotheses for osteoclast-targeted research. Current treatment should remain fracture risk–based rather than guided by an unvalidated turnover phenotype.

## 1 Introduction: the osteoclast paradox in type 2 diabetes

T2DM is now firmly established as a clinically relevant cause of skeletal fragility. A 2026 systematic review and meta-analysis incorporating more than 13 million participants confirmed increased fracture risk across several skeletal sites (1). Prospective data also demonstrate that conventional tools remain informative but systematically underrepresent the diabetic skeletal burden: for a given femoral-neck T-score or FRAX probability, older adults with T2DM experience more fractures than individuals without diabetes (2). Population studies from China and Korea similarly document vertebral and nonvertebral fracture burdens that are not adequately explained by low areal BMD (3, 4). The clinically important observation is that fracture risk may remain elevated even when DXA-derived areal BMD (aBMD) is not low (5); this dissociation does not by itself identify a single causal mechanism. This aBMD pattern should be interpreted as a population-level DXA phenotype rather than direct evidence of intrinsically stronger bone: obesity can increase skeletal loading and alter endocrine/adipokine signals, while surrounding soft tissue can introduce site- and configuration-dependent error and reduce DXA precision (6–8). Fracture risk in T2DM reflects both impaired skeletal competence and increased exposure to falls through neuropathy, visual impairment, hypoglycemia, sarcopenia, and frailty; this Review focuses primarily on skeletal mechanisms while recognizing their clinical interaction with extra-skeletal risk.

At the biochemical level, the dominant population-level pattern in T2DM is lower circulating concentrations of markers of both bone formation and resorption. The systematic review by Hygum et al. and the T2DM-specific meta-analysis by Yang et al. consistently reported lower osteocalcin, procollagen type I N-terminal propeptide (P1NP), and C-terminal telopeptide of type I collagen (CTX) (9, 10). The 2026 DiaFALL cohort independently reproduced the combination of higher aBMD and reduced intact P1NP, osteocalcin, and β-CTX-I (11). These findings support a reproducible low-BTM phenotype at the population level, but circulating marker concentrations should not be treated as direct measures of osteoclast number, mature resorptive function, or basic multicellular unit (BMU) kinetics. Diabetic skeletal fragility therefore cannot be explained simply by the high-turnover model that underlies much of conventional postmenopausal osteoporosis.

Direct human evidence that osteoclast-lineage biology is dissociated from circulating BTMs is limited. In a single exploratory study of 21 women with T2DM and 21 matched controls, osteoclast precursors were reported to be more numerous but less mature, with lower circulating RANKL (12); this finding has not been independently replicated and should not be generalized as an established T2DM phenotype. At the tissue level, trans-iliac histomorphometry suggests that remodeling units can remain coupled despite delayed progression through the reversal–resorption sequence (13). Together, these observations motivate—rather than establish—the hypothesis that precursor state, mature resorptive function, and whole-skeleton biochemical output may become dissociated in T2DM.

A separate clinical observation concerns antiresorptive therapy. Individual-participant data from 15 randomized osteoporosis trials did not identify clear attenuation of aBMD, BTM, or pooled fracture responses in participants with T2DM (14). However, treatment response is a clinical outcome rather than a direct assay of endogenous mature osteoclast resorptive function, and it should not be used as mechanistic proof of a distinct osteoclast phenotype. Instead, these data indicate that low baseline circulating turnover markers alone are insufficient to infer absence of benefit from antiresorptive therapy.

In this Review, the term “osteoclast paradox” is used as an author-proposed heuristic framework—not a diagnostic entity or a biologically validated phenotype—to organize apparent dissociations among circulating bone-turnover markers, osteoclast-lineage biology, tissue-level remodeling, matrix properties, and treatment response in T2DM. We do not assume that these observations necessarily coexist in the same individual or arise from a single causal mechanism. The novelty of this Review lies not in redefining T2DM as a low-turnover skeletal disorder, but in explicitly separating non-equivalent osteoclast-related levels of evidence—including circulating resorption markers, precursor state, differentiation and fusion, mature resorptive function, BMU kinetics, matrix degradability, and therapeutic responsiveness—and organizing them within a testable multiscale framework. The purpose is to distinguish established observations from indirect mechanistic inferences and hypotheses, and to define the human studies needed to connect these levels.

### 1.1 Literature search, study selection, and evidence synthesis

This mechanism-oriented narrative Review used a structured but non-systematic evidence-identification approach. PubMed/MEDLINE was searched from database inception to 28 July 2026, supplemented by backward and forward citation tracking and targeted searches of journal and publisher websites. The original search was iterative and organized around prespecified concept blocks covering the T2DM skeletal phenotype, osteoclast lineage and function, bone turnover and histomorphometry, bone quality and microarchitecture, osteocyte/BMU biology, metabolic and vascular modifiers, antiresorptive and osteoanabolic therapies, treatment sequencing, glucose-lowering therapies, and osteoclast-targeted drug discovery. we therefore did not retrospectively construct a PRISMA flow diagram or pseudo-precise screening counts. For transparency and reproducibility, the concept blocks were translated during revision into explicit PubMed search syntax, reported in **Supplementary Table S1**.

Eligibility was defined by relevance to adult T2DM skeletal biology or treatment. Human cohort, case-control, cross-sectional, bone-biopsy/histomorphometric, human cellular, randomized-trial, individual-participant, systematic-review/meta-analytic, and major guideline evidence was prioritized. Preclinical animal and *in vitro* studies were included only when they addressed mechanistic questions that could not be resolved directly in humans, such as differentiation-stage effects, matrix degradability, cytoskeletal organization, or osteoclast–osteoblast coupling. Studies restricted to type 1 or gestational diabetes, pediatric populations, non-peer-reviewed conference abstracts, or reports without a directly relevant skeletal, remodeling, osteoclast-lineage, bone-quality, fracture, or treatment endpoint were not used to support core claims. No database-level language filter was applied; however, retained studies required sufficient English-language abstract or full-text information to permit appraisal of design, population, methods, and outcomes. When multiple publications arose from the same cohort or trial, the most informative report for the question of interest was prioritized, while companion analyses were retained when they contributed a distinct endpoint.

Study selection and interpretation were question-specific rather than based on a single universal evidence hierarchy. For population-level phenotype and clinical treatment claims, direct human T2DM data, adequately characterized cohorts, randomized comparisons, and replicated findings were given greatest weight. For mechanistic claims, direct human tissue or cellular evidence was distinguished from animal or *in vitro* evidence, which was used primarily to establish biological plausibility. Across study types, greater weight was given to larger samples, standardized assays or imaging, explicit control of major confounders, directness of the measured endpoint, independent replication, and consistency with complementary evidence. Contradictory findings were retained and discussed rather than selectively excluded. Studies were selected for the evidence tables when they directly informed a central claim, represented an important counterexample, or materially affected clinical interpretation, and when sufficient methodological detail was available to define the population, design, endpoint, and major limitation. Evidence extrapolated from the general osteoporosis population is identified separately from T2DM-specific evidence.

## 2 Defining the low-BTM phenotype in type 2 diabetes

### 2.1 What do circulating bone turnover markers actually measure?

The most reproducible biochemical feature of T2DM is a population-level pattern of lower circulating bone-turnover-marker concentrations. Meta-analytic and cohort data consistently report lower P1NP, osteocalcin, and CTX (as labeled in the source studies) (9–11), with similar findings in postmenopausal case-control cohorts (15, 16). Because markers of both formation and resorption are generally lower, this pattern is compatible with reduced average remodeling output. However, circulating concentrations alone do not quantify BMU activation frequency, osteoclast number, or mature cellular resorptive competence.

Circulating BTMs are biochemical outputs rather than direct cellular or histomorphometric measurements. The IOF/IFCC reference resorption marker is β-isomerized C-terminal telopeptide of type I collagen (β-CTX-I), which reflects type I collagen degradation and is sensitive to preanalytic and biological factors including sampling time, fasting status, renal function, and assay conditions (17). It is not a direct assay of osteoclast number or pit-resorptive capacity. TRACP nomenclature also requires assay-level precision: total tartrate-resistant acid phosphatase (TRACP) and the osteoclast-associated isoform TRACP5b are not interchangeable. The 2024 meta-analysis reported lower CTX but no significant difference in pooled TRACP as labeled by the included source studies (10); those pooled TRACP data should not be reinterpreted as TRACP5b. In this Review, CTX is retained when a source publication reports the marker generically, whereas β-CTX-I is used when the assay is specified or when referring to the standardized reference marker.

Accordingly, four measurement levels are kept distinct throughout this Review: (i) circulating biochemical output, including P1NP, osteocalcin, and CTX/β-CTX-I; (ii) osteoclast-lineage state, including precursor abundance, commitment, fusion, and differentiation; (iii) mature osteoclast functional competence, including polarization, acidification, sealing-zone formation, and pit or matrix resorption; and (iv) tissue-level remodeling, including basic multicellular unit (BMU) activation and reversal–resorption–formation kinetics. Evidence at one level should not be treated as proof of another.

### 2.2 Histomorphometry: low turnover is not absent turnover

Histological evidence, although much smaller in scale than BTM studies, provides essential mechanistic resolution. Early work documented depressed formation indices in diabetes (18). Manavalan et al. later demonstrated fewer circulating osteogenic precursors and, in a small biopsy subset, reduced mineralizing surface, bone formation rate, and osteoblast surface (19). These observations are consistent with a formation defect, but they do not support complete remodeling arrest.

The 2025 trans-iliac biopsy study is particularly informative because it directly examined cortical remodeling states in longstanding T2DM. Cortical pore area, mean pore size, and pore density were not increased, but the distribution of remodeling states shifted toward eroded-formative pores, consistent with prolonged progression through the reversal–resorption period (13). The phrase “delayed but coupled” is therefore more precise than either “high resorption” or “osteoclast shutdown.” These tissue findings demonstrate ongoing coupled remodeling in the sampled iliac cortex despite a low circulating-BTM phenotype; they do not quantify whole-skeleton BMU activation frequency or establish preserved mature osteoclast function throughout the skeleton.

### 2.3 Osteoclast precursor–mature osteoclast dissociation

The Sassi study is one of the few direct human investigations of osteoclast-lineage phenotype in T2DM. In 21 women with T2DM and 21 matched controls, osteoclast precursors were reported to be more numerous but less mature, osteoblast precursors were reduced, and circulating RANKL was lower (12). This single small study supports a possible precursor–mature-function dissociation, but it has not been independently replicated and should not be generalized as a population-level T2DM phenotype or used as direct evidence of tissue-level resorptive activity.

### 2.4 Low turnover is metabolically heterogeneous

Low turnover cannot be reduced to mean glycated hemoglobin (HbA1c) or disease duration. Continuous-glucose-monitoring data show that glycemic excursions and dawn glucose relate inversely to CTX and P1NP (20), suggesting that dynamic glycemia may carry information not captured by a single glycated hemoglobin measurement. Conversely, the DiabOS cohort identified parathyroid hormone (PTH) and mineral-metabolism variables as important determinants of turnover, while HbA1c and duration were not independently dominant after adjustment (21).

Crucially, low formation is already present near diagnosis. Postmenopausal women with newly diagnosed, treatment-naïve T2DM had lower P1NP and osteocalcin (22). In a larger cohort of 326 drug-naïve patients, low-turnover patterns differed across metabolic phenotypes, with insulin-resistant/hyperinsulinemic phenotypes showing lower P1NP, CTX, and osteocalcin than relatively insulin-sensitive insulinopenic phenotypes (23). These data undermine an attractive but unsupported narrative in which early diabetes is necessarily high-turnover and only longstanding disease becomes suppressed.

A more plausible model is that remodeling phenotype emerges from interacting metabolic dimensions: insulin sensitivity, endogenous insulin availability, glycemic variability, renal function, PTH, obesity, sex, menopausal status, complications, physical activity, and treatment exposure. Disease duration influences these domains but cannot substitute for them.

### 2.5 Obesity, body composition, and metabolic confounding

Obesity is common in T2DM but is not biologically interchangeable with diabetes, insulin resistance, or hyperglycemia. Higher body mass can increase DXA-derived aBMD through greater mechanical loading and endocrine or adipokine effects, while obesity itself has site- and sex-dependent associations with fracture risk and bone microarchitecture (8). In addition, experimental fat-layering studies show that surrounding adipose tissue can alter the accuracy and precision of DXA measurements; importantly, the direction and magnitude of this error depend on skeletal site, body-composition configuration, and scanner conditions, so higher aBMD in T2DM should not be dismissed as a uniform measurement artifact (6, 7).

The overlap among obesity, insulin resistance, hyperinsulinemia, and hyperglycemia complicates causal attribution. A study restricted to non-obese postmenopausal women still found lower P1NP/β-CTX-I and greater fracture burden in T2DM, suggesting that obesity does not fully explain the biochemical or clinical phenotype, although the cross-sectional design limits causal inference (16). Accordingly, studies of diabetic bone should report and, where possible, account for BMI, regional adiposity or body composition, insulin sensitivity, glycemic exposure, physical loading, and weight change rather than treating “T2DM” as a single metabolic exposure.

### 2.6 Sclerostin: plausible contributor, insufficient single explanation

Several human studies report higher circulating sclerostin in T2DM and associations with lower turnover or higher DXA-derived aBMD (24, 25). Selected cohorts have also linked higher sclerostin with vertebral fracture independently of aBMD and conventional BTMs (26). These observations are biologically compatible with impaired Wnt signaling and reduced formation, but circulating sclerostin is not a direct readout of local osteocyte signaling.

A 2025 multicenter analysis measuring total and bioactive sclerostin showed that estimated glomerular filtration rate (eGFR) was the strongest independent determinant and did not reproduce consistent associations with BTMs after adjustment (27). Therefore, sclerostin should be treated as one component of an altered osteocyte environment rather than as the singular causal explanation for diabetic low turnover.

Taken together, human studies support a reproducible low-BTM biochemical phenotype in T2DM that is compatible with reduced average remodeling output, while direct evidence for cell-specific osteoclast function and tissue-level BMU kinetics remains much more limited. The distinction between these measurement levels is central to interpreting apparent discordances in the literature.

Representative human evidence defining the low-turnover and osteoclast phenotype is summarized in **Table 1**.

**Table 1:** Representative human evidence relevant to the low-BTM phenotype and osteoclast-related biology in type 2 diabetes mellitus.

Study/sample	Population/design	Key finding	Evidence type/contribution	Main limitation
Shu et al., 2012 (15) 25 T2DM + 25 controls	Postmenopausal women; case-control; HR-pQCT/BTMs	Lower P1NP and osteocalcin with largely preserved structure	Human observational (case-control) — clinical low-BTM phenotype	Cross-sectional; small sample; HR-pQCT subset of 14 pairs.
Jiajue et al., 2014 (16) n = 1, 410	Postmenopausal Chinese women stratified by BMI and glycemic status	Lower P1NP/β-CTX-I and higher fracture burden in non-obese T2DM despite non-low aBMD	Human observational (population study) — low-BTM/fracture association	Cross-sectional fracture assessment; population-specific.
Manavalan et al., 2012 (19) 18 T2DM + 27 controls; histomorphometry n = 9	Postmenopausal women; circulating osteogenic precursors + tissue subset	Fewer osteogenic precursors and lower formation indices	Human cellular + tissue subset — direct formation/tissue evidence	Small tissue subset; primarily informs the formation arm rather than osteoclast function.
Sassi et al., 2018 (12) 21 T2DM + 21 matched controls	Postmenopausal women; circulating precursor phenotyping	More but less mature osteoclast precursors; fewer osteoblast precursors; lower RANKL	Human cellular (exploratory) — possible osteoclast-lineage dissociation	Small postmenopausal cohort; single study; not independently replicated; no direct tissue-level resorption measurement.
Starup-Linde et al., 2021 (20) 100 T2DM + 100 controls recruited; 196 analyzed	T2DM with 3-day continuous glucose monitoring	Greater glycemic variability/dawn glucose associated with lower CTX/P1NP	Human observational (CGM cohort) — glycemic-variability modifier	Observational association; short CGM window.
Vavanikunnel et al., 2022 (21) 110 T2DM + 92 controls	DiabOS multicenter baseline cohort	PTH/mineral variables associated with low CTX/P1NP	Human observational (multicenter cohort) — PTH/renal–mineral modifier	Cross-sectional; demographic imbalance and residual confounding.
Nasser et al., 2023 (23) n = 326	Newly diagnosed, drug-naïve T2DM phenotypes	Lower BTMs in insulin-resistant/hyperinsulinemic phenotypes	Human observational phenotyping — metabolic heterogeneity	Phenotypes defined by surrogate estimates; no control or fracture endpoint.
Andreasen et al., 2025 (13) 30 T2DM + 10 controls analyzed	Trans-iliac cortical bone histomorphometry	Delayed but coupled remodeling; no universal excess cortical porosity	Human histomorphometric tissue study — direct BMU-state evidence	Small cohort; mainly older adults; limited generalizability.
Rasmussen et al., 2026 (11) 105 T2DM + 105 matched controls	DiaFALL matched cross-sectional cohort	Higher DXA-derived aBMD with broadly suppressed bone-status indices	Human observational matched cohort — independent biochemical-phenotype replication	Single center; cross-sectional; moderate sample.

aBMD, areal bone mineral density; BTM, bone turnover marker; CTX, C-terminal telopeptide of type I collagen as reported by source studies; HR-pQCT, high-resolution peripheral quantitative computed tomography; P1NP, procollagen type I N-terminal propeptide; PTH, parathyroid hormone; RANKL, receptor activator of nuclear factor-κB ligand; T2DM, type 2 diabetes mellitus. β-CTX-I denotes the β-isomerized C-terminal telopeptide reference marker when assay specificity is established.

## 3 How the diabetic milieu reprograms osteoclast biology

### 3.1 Hyperglycemia: substrate, stressor, and context modifier

Osteoclast differentiation itself is metabolically dynamic; early RANKL stimulation increases glucose metabolism in precursor cultures (28). Evidence attributed specifically to hyperglycemia, however, comes from non-equivalent experimental systems. In RAW264.7 cells, high glucose suppressed RANKL-induced differentiation and reduced the fusion-related molecules ATP6V0D2 and DC-STAMP (29). In primary C57BL/6 bone-marrow monocytes, 33.6 mmol/L glucose, with or without 1 μmol/L insulin, reduced osteoclast formation and pit resorption (30). A rat T2DM alveolar bone-defect model and matched ex vivo assays likewise showed reduced osteoclast differentiation, resorptive function, and matrix degradation during bone regeneration (31). These models support direct inhibitory effects under defined conditions, but they differ substantially in glucose concentration, insulin exposure, cell source, skeletal site, and whether the endpoint is steady-state remodeling or repair.

Other models demonstrate the opposite direction. Exposure to 25 mmol/L glucose and streptozotocin-induced hyperglycemia increased osteoclastogenesis and bone resorption through CaMKII signaling in rats (32). Because streptozotocin produces an insulin-deficient phenotype and the study itself is framed as a type 1 diabetes model, it should be interpreted as evidence that hyperglycemia can promote osteoclastogenesis under a specific metabolic context, not as direct evidence for a high-resorption phenotype in typical T2DM. Catalfamo et al. combined db/db mouse cultures with peripheral-blood-derived human osteoclasts in a periodontal/inflammatory context; human T2DM-derived cells showed similar baseline differentiation and resorptive capacity to controls but altered responses to lipopolysaccharide (33). This finding supports context-dependent inflammatory responsiveness rather than generalized elevation of basal osteoclast function in T2DM.

### 3.2 AGEs–RAGE signaling: the cell is abnormal, but so is the substrate

Advanced glycation end product (AGE) biology also derives from heterogeneous models. RAGE-deficient mice and murine bone-marrow macrophages demonstrate that RAGE participates in osteoclast maturation, cytoskeletal organization, and resorptive function, but this is a genetic loss-of-function model rather than a diabetic model (34). *In vitro* glycation of bone or ivory matrices reduces degradation by mature osteoclasts, while exogenous AGE-modified proteins can inhibit osteoclastogenesis (35). In RAW264.7-derived osteoclast-like cells, AGE effects vary by differentiation stage, with inhibition during fusion but modest enhancement of resorption when exposure occurs after maturation (36). Glycolaldehyde-modified AGE-BSA also suppresses differentiation of CD14-positive monocytes from healthy human donors through an IL-10-associated mechanism (37), and marrow-derived macrophage experiments report suppression of both osteoclast- and osteoblast-lineage differentiation by AGEs (38).

Collectively, these experiments establish biological plausibility for both cell-intrinsic and substrate-mediated AGE effects, but none reproduces the full human T2DM bone microenvironment. AGE preparation, concentration, cell source, matrix substrate, and differentiation stage materially influence the observed direction of effect. The hypothesis that glycated matrix contributes to lower collagen-fragment release *in vivo* therefore remains unproven; no human study has quantified the contribution of matrix resistance to circulating CTX independently of BMU activation and osteoclast number.

### 3.3 Oxidative stress and mitochondrial competence

Controlled reactive oxygen species participate in physiological RANKL signaling. Nrf2-deficient mouse osteoclast precursors show increased ROS, enhanced differentiation, actin-ring formation, and resorption (39), demonstrating that redox state can regulate osteoclastogenesis. This genetic redox model is not a model of T2DM, however, and cannot establish the net effect of chronic diabetic oxidative stress on mature human osteoclast competence. A stage-dependent interpretation—in which moderate redox signaling may facilitate differentiation whereas sustained metabolic injury may impair mitochondrial, cytoskeletal, or vesicular function—remains biologically plausible but requires direct testing in human T2DM-derived cells.

### 3.4 Insulin signaling and insulin resistance

Insulin effects likewise depend on experimental system and lineage stage. In RANKL-stimulated precursor cultures, insulin can enhance osteoclastogenesis through ERK1/2, NFATc1, and ATP6V0D2 signaling (40), whereas primary and cultured murine/rat osteoclast-like cells can show insulin-mediated inhibition of pit formation (41). Osteoblast insulin signaling can also regulate osteoclast-dependent resorption and systemic energy metabolism in genetic mouse models (42). These studies demonstrate that insulin can influence several components of the remodeling network, but they do not establish how hyperinsulinemia, insulin resistance, β-cell failure, or exogenous insulin therapy alters osteoclast function in human T2DM. Those metabolic states should therefore not be treated as interchangeable exposures.

### 3.5 Inflammation, marrow stromal signals, and local niches

Inflammatory and stromal signals require similar separation by model. Genetic mouse studies show that bone-marrow adipose-lineage cells can provide RANKL for trabecular osteoclast formation and pathological bone loss (43), but this finding is not T2DM-specific and the effect is compartment- and challenge-dependent. Human marrow-fat studies, by contrast, are observational and show sex-, site-, and lipid-dependent heterogeneity rather than a universal increase in marrow adiposity in T2DM (44, 45). Periodontal studies provide information about a specialized oral inflammatory microenvironment (46), but they should not be used as evidence that comparable focal osteoclast activation is generally present in the axial or appendicular skeleton. Marrow adiposity and local inflammation are therefore treated as contextual modifiers, not established primary drivers of the diabetic low-turnover phenotype.

### 3.6 Microvascular disease and perfusion

Microvascular disease may alter skeletal perfusion, oxygen tension, osteocyte viability, and repair. In a combined high-resolution peripheral quantitative computed tomography (HR-pQCT) and dynamic contrast-enhanced magnetic resonance imaging (DCE-MRI) study, microvascular disease rather than T2DM itself was independently associated with higher cortical porosity and altered intracortical vessel characteristics (47). This observational association does not establish that vascular disease causes porosity or that osteoclasts mediate the relationship.

A plausible remodeling-network link is nevertheless testable: impaired perfusion and local hypoxia could compromise osteocyte viability or damage sensing, which in turn may alter local RANKL/OPG signaling and recruitment of a basic multicellular unit. Experimental T2DM models showing abnormal osteocyte calcium dynamics and mechano-responsiveness are compatible with such a pathway (48), but direct human evidence connecting microvascular dysfunction to osteocyte signaling, site-specific osteoclast recruitment, and subsequent remodeling remains lacking. We therefore treat vascular disease as a contextual modifier of skeletal heterogeneity rather than an established osteoclast driver.

### 3.7 Glucose-lowering and weight-loss therapies as phenotype modifiers

Medication exposure is another major source of biological and epidemiological heterogeneity. Thiazolidinediones have a reproducible adverse skeletal signal, with increased fracture risk—particularly in women—and reductions in aBMD reported in randomized-trial meta-analyses (49). By contrast, pooled data for SGLT2 inhibitors have not shown a consistent increase in fracture risk or clinically important reduction in aBMD (50, 51), and observational meta-analysis has not demonstrated a robust fracture-risk reduction with metformin (52). Evidence for GLP-1 receptor agonists is evolving; effects on body weight, lean mass, glycemic control, falls, and potentially bone turnover can change the skeletal context even when a direct osteoclast effect is uncertain (53–55).

Insulin therapy can additionally mark more advanced disease while increasing hypoglycemia-related fall exposure, creating confounding by indication when fracture outcomes are interpreted. Pharmacological or surgical weight loss can alter mechanical loading and body composition independently of glucose lowering. Consequently, mechanistic and clinical T2DM studies should document glucose-lowering class, duration of exposure, recent weight change, hypoglycemia, and prior osteoporosis therapy. Medication exposure is treated here as a modifier and potential confounder of the observed skeletal phenotype, not as evidence for a single osteoclast mechanism.

Taken together, experimental findings should not be collapsed into a single ‘diabetic osteoclast’ effect. Species, cell source, diabetes model, glucose and insulin exposure, AGE preparation, differentiation stage, skeletal site, inflammatory context, and the functional assay all influence the observed phenotype. Human T2DM-derived cellular data remain limited, whereas many mechanistic studies isolate one exposure under conditions that do not reproduce the integrated metabolic state of T2DM.

### 3.8 Interpreting model heterogeneity and translational limits

Accordingly, apparently contradictory results across Sections 3.1–3.5 are better interpreted as model-dependent effects than as evidence for a universal switch between pro- and anti-osteoclastogenic states. Direct human evidence is given the greatest weight for claims about the T2DM phenotype; animal and cell models are used to identify plausible mechanisms, not to infer their prevalence or net direction in patients. **Supplementary Table S2** summarizes the principal experimental systems, metabolic context, osteoclast endpoint, and translational limitation of the mechanistic studies used in this Review.

## 4 Reconciling osteoclast-related endpoints across measurement levels

### 4.1 From pathway activation to whole-skeleton turnover

The phrase “osteoclast activity” is often used for non-equivalent endpoints and is therefore avoided unless the underlying measurement is specified. NF-κB or NFATc1 activation indicates intracellular signaling; TRAP-positive multinucleated cells indicate differentiation; precursor counts indicate lineage abundance; sealing-zone formation and pit assays assess mature functional competence; CTX/β-CTX-I reflects collagen-degradation products in the circulation; and histomorphometry characterizes tissue-level remodeling states (10, 12, 17). A change at one level cannot be assumed to determine another.

The osteoclast life cycle contains several potential bottlenecks, including recruitment, RANKL-dependent commitment, fusion, polarization, acidification, matrix degradation, and coupling. The small exploratory human precursor study (12), high-glucose effects on fusion-related molecules (29), and stage-dependent AGE effects (35, 36) illustrate biologically plausible sources of dissociation across lineage stages. They do not, however, establish a generalized *in vivo* dissociation between precursor abundance and mature resorptive function in human T2DM.

### 4.2 Spatial and metabolic heterogeneity

Circulating CTX is a whole-skeleton biochemical output and cannot localize remodeling to a specific skeletal site. Human T2DM studies demonstrate site- and compartment-dependent differences in microarchitecture and tissue remodeling, including discordant cortical findings across the radius, tibia, and trans-iliac biopsy (13, 47, 56–61). These observations support skeletal heterogeneity, but they do not directly demonstrate systematic differences in mature osteoclast function among anatomical compartments. Specialized local inflammatory models, including periodontal studies, are therefore treated separately and are not used as evidence for generalized focal osteoclast activation elsewhere in the skeleton. Direct paired human evidence linking circulating BTMs to site-specific mature osteoclast function remains lacking.

Metabolic heterogeneity is better supported than compartment-specific osteoclast heterogeneity. Glycemic variability, PTH/mineral metabolism, and insulin-sensitivity phenotype are associated with turnover-marker differences, while low formation is already detectable in newly diagnosed untreated T2DM (20–23). These findings justify retaining metabolic phenotype as an analytic axis, but they do not establish a deterministic early-high/late-low sequence or a single osteoclast state shared across T2DM.

### 4.3 Matrix context as a second determinant of resorption

Matrix composition provides a second determinant of resorptive output. In T2DM, AGE-modified bone may alter the amount of collagen fragment released per remodeling event, although the *in vivo* contribution of this mechanism to circulating CTX remains unknown (35, 62, 63).

### 4.4 The four-axis framework

The four-axis framework organizes the literature along four analytic dimensions: lineage stage, skeletal site/anatomical compartment, metabolic phenotype, and remodeling/matrix context (12, 13, 23, 35, 47, 56–61). These axes should not be interpreted as four jointly validated components of a single T2DM phenotype. Evidence is strongest for the biochemical low-turnover phenotype and clinical skeletal heterogeneity, whereas direct human evidence for lineage-stage and compartment-specific osteoclast function remains sparse. The framework is intended to prevent non-equivalent endpoints—such as circulating CTX/P1NP concentrations, precursor phenotype, mature resorptive function, BMU kinetics, and matrix degradability—from being treated as interchangeable measures of ‘osteoclast activity.’

The framework therefore generates hypotheses rather than clinical classifications. Examples include whether patients with similar circulating BTM profiles differ in osteoclast-lineage state or ex vivo function; whether skeletal sites differ in remodeling behavior; whether glycated human bone alters collagen-fragment release under standardized resorption; and whether metabolic phenotypes modify BMU kinetics or mature osteoclast competence. These propositions have not been demonstrated together within the same T2DM population and require paired, within-participant studies integrating biomarkers, cellular assays, imaging, and, where feasible, tissue-level measurements. **Figure 1** illustrates this hypothesis-generating framework; it is not a validated patient classification or treatment-selection algorithm.

**Figure 1:**
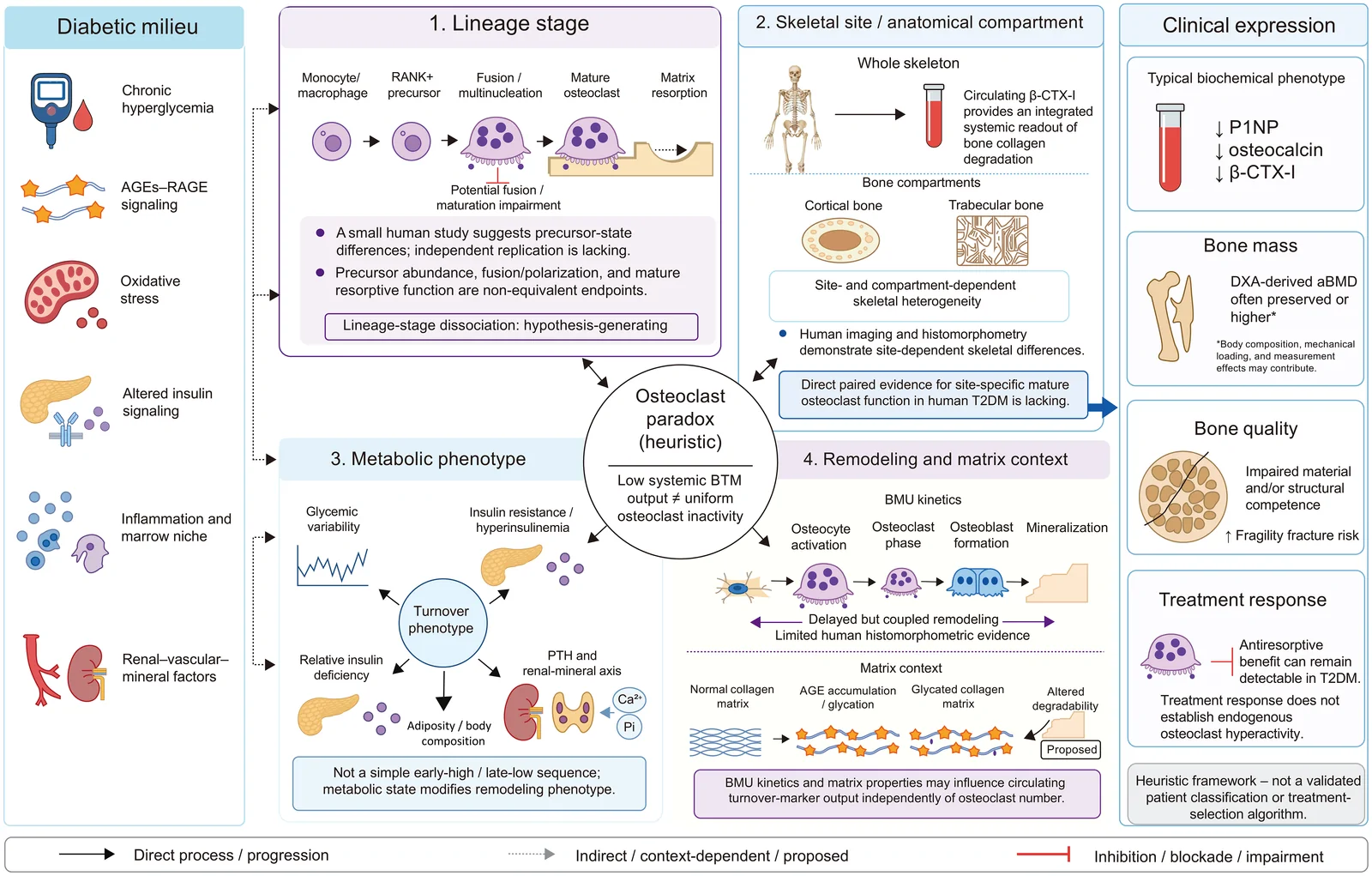
Four-axis heuristic framework for osteoclast-related dissociations in type 2 diabetes. The image distinguishes four analytic dimensions—osteoclast lineage stage, skeletal site/anatomical compartment, metabolic phenotype, and remodeling/matrix context—without assuming that they form a single validated T2DM phenotype. Evidence strength is deliberately shown as uneven: the lineage-stage axis is hypothesis-generating and relies on limited direct human data; skeletal-site heterogeneity is supported by imaging and histomorphometry but not by paired measurements of site-specific mature osteoclast function; and metabolic, BMU, and matrix effects remain context dependent. DXA-derived aBMD is presented as a measurement phenotype that may be influenced by body composition, loading, and soft-tissue effects. Antiresorptive treatment response is shown separately from mechanistic inference because clinical benefit does not establish endogenous osteoclast hyperactivity. The framework is not a patient classification or treatment-selection algorithm.

## 5 Why low bone turnover does not protect the diabetic skeleton

In this Review, “bone quality” is used as an umbrella term for skeletal properties not captured by bone mass alone, including tissue material behavior, microarchitecture, mineralization, microdamage distribution and repair, and tissue age. These components are not interchangeable, and no single circulating marker, imaging metric, or tissue assay directly measures bone quality as a whole.

### 5.1 Fracture occurs at higher aBMD and is incompletely captured by FRAX

The dissociation between bone quantity and fragility is clinically measurable. DXA-derived aBMD and FRAX remain predictive in T2DM, but at any given femoral-neck aBMD or FRAX estimate the observed fracture risk is higher than in people without diabetes (2, 64). Longer diabetes duration further increases risk and worsens underestimation by FRAX (65). Thus, a non-low aBMD does not imply preserved mechanical competence and should not be equated with normal bone quality. Conversely, the higher or preserved DXA-derived aBMD reported in many T2DM cohorts should not be interpreted as a pure biological increase in bone strength because obesity-related loading and soft-tissue measurement effects can contribute to the observed value (6–8).

Trabecular bone score (TBS) provides complementary information. Large registry data and meta-analysis show lower TBS on average in T2DM despite preserved or increased DXA-derived aBMD (66, 67). TBS adjustment contributes fracture-risk information beyond conventional FRAX variables (68). TBS should not be misrepresented as direct histology, but it supports the broader conclusion that spatial organization and tissue quality are not fully represented by areal density.

### 5.2 AGE accumulation and tissue material properties

AGE accumulation is one of the most plausible links between chronic metabolic exposure and impaired tissue quality. Human microindentation studies have demonstrated lower material strength in postmenopausal T2DM (69), and greater AGE burden is associated with lower material strength and lower formation markers (62). Additional human work links AGE accumulation and tissue oxygenation to material properties and cortical porosity (70).

Direct analysis of hip-fracture tissue demonstrates increased nonenzymatic cross-linking and altered post-yield mechanical behavior in diabetic trabecular bone (63). These findings fit the concept that glycation reduces collagen molecular mobility and energy dissipation. However, the relationship between composition and mechanical failure is not uniform.

The 2024 multiscale study by Britton et al. is important because it provides counter-evidence. Diabetic specimens showed increased glycation products and altered mineral/matrix composition but did not show deterioration in every nano- or macro-mechanical endpoint (71). Rather than weakening the field, this inconsistency suggests that skeletal site, fracture status, loading mode, tissue age, sex, and specimen selection determine which material deficits are detectable.

### 5.3 Microdamage: quantity may be less informative than distribution and repair

Suppressed remodeling is often assumed to cause microcrack accumulation. Direct human evidence is more nuanced. Sacher et al. did not find a universal increase in total microdamage but observed altered distribution between trabecular rods and plates (72). Britton et al. similarly did not demonstrate generalized excess microdamage (71). The relevant abnormality may therefore involve where damage occurs, how it propagates, and whether remodeling is appropriately targeted to damaged regions rather than simply how many cracks are present.

The relevance of microdamage to the present framework is not that microcrack burden proves osteoclast dysfunction. Rather, repair of mechanically relevant damage depends on osteocyte sensing, local BMU recruitment, osteoclast-mediated removal of damaged matrix, and subsequent formation. Thus, altered damage distribution or inefficient damage-targeted remodeling could matter even without a generalized increase in total histological microcrack count.

### 5.4 Mineralization and microarchitecture: no single diabetic structural phenotype

Low turnover is also frequently equated with hypermineralization. Human tissue studies do not support a uniform direction of mineral-to-matrix change (63, 71). The safer concept is abnormal mineral–matrix integration rather than universal excessive mineralization.

HR-pQCT studies illustrate the same heterogeneity. Early work by Burghardt et al. demonstrated compartment-specific microarchitectural differences in women with T2DM despite preserved areal BMD (56). Patsch et al. found strikingly higher cortical porosity in T2DM women with fragility fractures compared with T2DM women without fracture (57). Yu et al. identified higher radial cortical porosity and lower cortical density in African-American women with T2DM (58), while the Framingham study identified lower cortical density, higher porosity, and smaller cross-sectional area at the tibia but not uniformly at the radius (59).

Yet other cohorts show apparently favorable architecture. Nilsson et al. reported greater trabecular bone volume fraction, larger cortical area, and higher estimated failure load in elderly women with T2DM, while bone material strength remained lower (60). More recent evidence attributes some porosity to microvascular disease rather than diabetes itself (47), and trans-iliac histomorphometry found no universal excess porosity (13).

Longitudinal data are sparse but informative. A five-year HR-pQCT follow-up demonstrated that changes in microarchitecture and estimated strength differ across diabetic fracture phenotypes, underscoring that a cross-sectional scan may capture only one stage of a dynamic process (61). The most defensible synthesis is therefore that cortical and trabecular abnormalities are subgroup-, site-, and method-dependent. There is no single structural signature that explains fracture risk in every person with T2DM.

### 5.5 Failure of tissue renewal as an integrative hypothesis

Normal remodeling performs two functions: regulating the amount of bone and renewing tissue that has accumulated metabolic aging or mechanical damage. In T2DM, the second function may be disproportionately important. Suppressed remodeling can preserve mineral mass while increasing the average residence time of collagen exposed to glycation, oxidative modification, and repeated loading.

This does not prove that low turnover is itself harmful. A causal longitudinal sequence—low turnover causing tissue aging, which then causes fracture—has not been demonstrated in humans. The more cautious interpretation is that reduced tissue renewal may amplify the consequences of an already abnormal metabolic matrix. This distinction is essential when considering whether further antiresorptive therapy is rational.

The multiscale pathways through which low remodeling may coexist with impaired bone quality are summarized in **Figure 2**.

**Figure 2:**
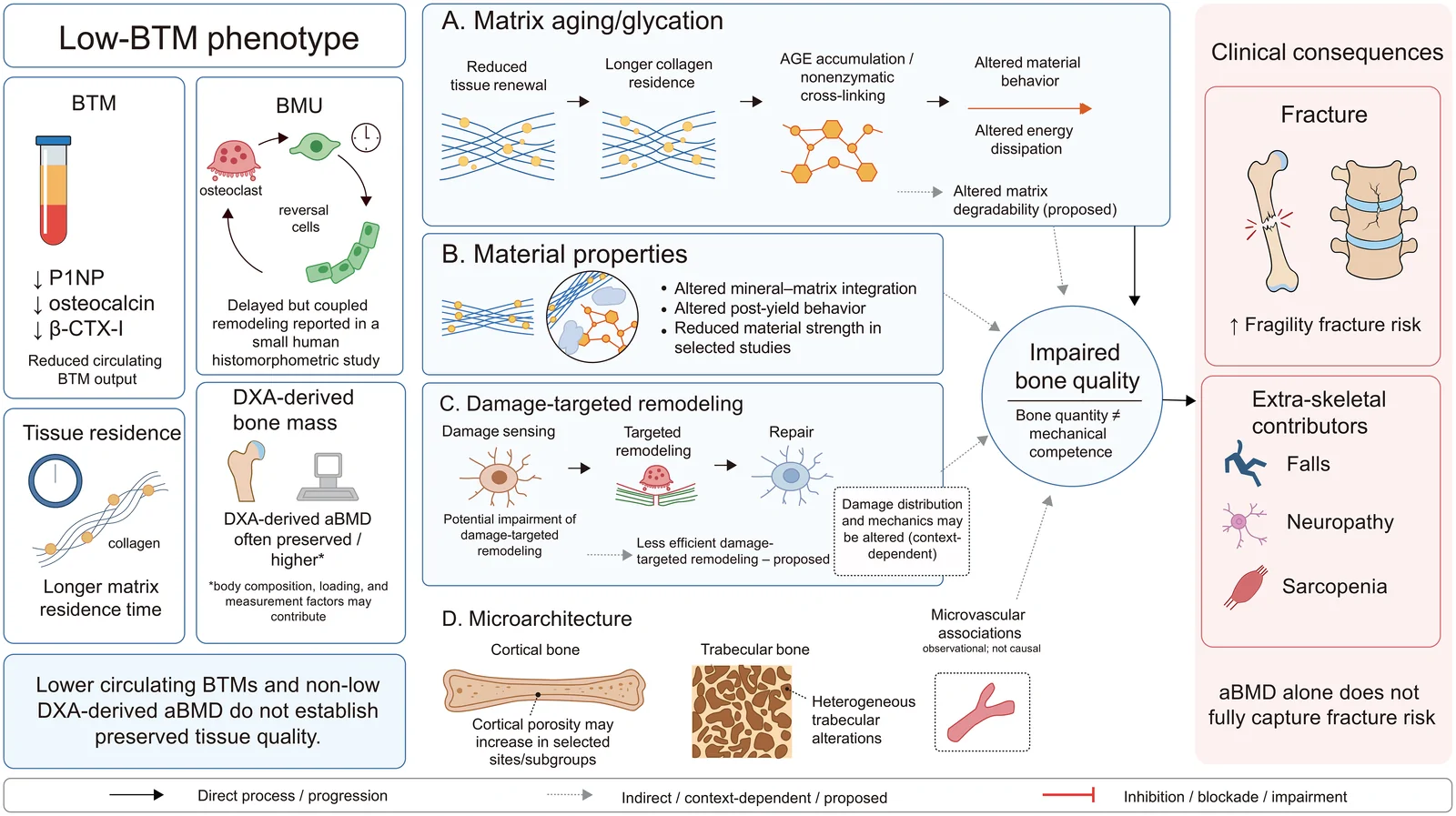
Why a low-BTM phenotype does not guarantee skeletal protection. Lower circulating BTMs are biochemical observations and should not be equated directly with tissue-level remodeling rate. They may coexist with non-low DXA-derived aBMD while tissue renewal, matrix composition, material behavior, damage-targeted remodeling, and microarchitecture remain abnormal or heterogeneous. DXA-derived aBMD may itself be influenced by obesity, mechanical loading, body composition, and soft-tissue measurement effects. In the image “bone quality” is an umbrella label for material properties, microarchitecture, mineralization, microdamage distribution/repair, and tissue age rather than a single directly measured variable. Delayed coupled remodeling derives from limited histomorphometric evidence, cortical porosity is site- and subgroup-dependent, and microvascular findings are observational associations rather than proof of causation. Generalized microdamage accumulation and uniform hypermineralization have not been consistently demonstrated in human T2DM bone. Extra-skeletal contributors such as falls, neuropathy, and sarcopenia/diabetes-related complications amplify observed fracture risk but are not direct measures of bone quality.

## 6 Osteoclast–osteoblast–osteocyte coupling in T2DM: a remodeling-network disorder

### 6.1 Osteocytes and remodeling initiation

A basic multicellular unit is not initiated by an osteoclast acting in isolation. Osteocytes form the principal mechanosensory network within mineralized bone and help coordinate remodeling through at least two complementary signaling axes: RANKL/OPG regulation of osteoclast recruitment and sclerostin/Wnt signaling that constrains osteoblast-lineage formation. Damage sensing and mechanical unloading can therefore influence both the resorptive and formative arms of a remodeling event.

In diabetes, several observations are compatible with osteocyte dysfunction, although the evidence spans different levels. Circulating sclerostin is often higher in T2DM, but associations with turnover are inconsistent after accounting for renal function (24–27). Experimental T2DM models demonstrate abnormal osteocyte calcium dynamics and impaired mechano-responsiveness (48), supporting a plausible defect in translating mechanical or damage signals into remodeling recruitment. These findings do not yet demonstrate that osteocyte dysfunction is the proximal cause of low circulating BTMs or fracture in humans with T2DM.

Vascular and osteocyte pathways may also intersect. Reduced perfusion or altered intracortical vascular characteristics could modify oxygenation and osteocyte viability, while impaired osteocyte signaling could alter the spatial recruitment of osteoclasts and osteoblasts around damaged tissue. The available human evidence is associative rather than causal (47), so this vascular–osteocyte–BMU pathway is best regarded as a mechanistic hypothesis requiring paired imaging, osteocyte, and tissue-remodeling measurements.

### 6.2 Formation and osteoclast-derived coupling

Human studies show abnormalities within the formation arm itself: circulating osteogenic precursors and biopsy-based formation indices are reduced, and osteoblast precursors can be diminished alongside immature osteoclast precursors (12, 19). The reduction in P1NP therefore cannot be assumed to be only a passive response to reduced resorption.

Osteoclasts also provide coupling signals. Human bone biopsy transcriptomics after denosumab-mediated suppression identified candidate osteoclast-derived factors including LIF, CREG2, CST3, CCBE1, and DPP4 (73). These factors are not established T2DM abnormalities, but they demonstrate that osteoclast-targeted interventions can influence subsequent formation beyond changing cavity depth.

### 6.3 The reversal phase and BMU kinetics

Human histomorphometry suggests modest prolongation of the reversal–resorption phase in T2DM (13), supporting slower BMU progression rather than remodeling arrest. Long-term alendronate treatment can also prolong this phase in human cortical bone (74); this comparison does not imply identical mechanisms, but illustrates how low turnover can arise from altered BMU kinetics rather than complete absence of osteoclasts.

### 6.4 A remodeling-network interpretation

Together, these findings support T2DM as a disorder of remodeling coordination: precursor pools can remain available, individual osteoclasts can be active, formation can be impaired, and the number or speed of BMUs can still decline. Metabolic low turnover is therefore not equivalent to endogenous pharmacological antiresorption.

## 7 Clinical antiresorptive evidence in T2DM: what treatment response does—and does not—show

### 7.1 Pooled randomized evidence: no detected effect modification is not proof of equivalence

Eastell et al. analyzed individual-participant data from 15 randomized antiresorptive trials comprising 96, 385 participants, including 6, 539 with T2DM for nonvertebral, all-fracture, and hip-fracture analyses and 3, 787 with T2DM for morphometric vertebral-fracture analyses (14). In participants with T2DM, the pooled treatment estimates were OR 0.48 (95% CI 0.36–0.63) for vertebral fracture, HR 0.86 (0.74–1.00) for nonvertebral fracture, HR 0.74 (0.64–0.84) for all fractures, and HR 0.82 (0.57–1.16) for hip fracture. The corresponding treatment-by-diabetes interaction p values were 0.53, 0.58, 0.80, and 0.33. In the surrogate-outcome analyses, the active-minus-placebo difference in total-hip aBMD change at 24 months was 3.86% (95% CI 3.54–4.18) in T2DM (interaction p=0.95), while CTX changed by −53.2% (95% CI −59.5 to −45.9) at 3–12 months (interaction p=0.69). These analyses show no statistically detectable attenuation of pooled treatment effects by T2DM; they do not establish formal equivalence, and precision differs by endpoint.

The clinically defensible inference is therefore narrower than “equivalent efficacy.” Low baseline circulating BTMs do not identify a therapeutic floor below which antiresorptive treatment becomes ineffective, but the pooled analysis combines drug classes and cannot establish identical effects for each agent or fracture endpoint. Treatment response is also a clinical observation, not a direct assay of endogenous mature osteoclast resorptive function.

### 7.2 Bisphosphonates: the most consistent class-specific evidence

Within the nine bisphosphonate trials included in the individual-participant analysis, the T2DM treatment estimates were OR 0.38 (95% CI 0.23–0.64; 74/1, 060 with vertebral fracture) for vertebral fracture, HR 0.77 (0.56–1.06; 152/1, 476) for nonvertebral fracture, HR 0.64 (0.49–0.83; 217/1, 476) for all fractures, and HR 1.13 (0.58–2.20; 37/1, 476) for hip fracture; none of the treatment-by-diabetes interactions was statistically significant (interaction p=0.21, 0.51, 0.32, and 0.14, respectively) (14). At 24 months, the active-minus-placebo total-hip aBMD difference in the T2DM bisphosphonate subset was 4.08% (95% CI 3.42–4.75; interaction p=0.20), while CTX suppression at 3–12 months was −51.1% (95% CI −59.8 to −40.5; interaction p=0.88). The wide hip-fracture confidence interval illustrates why absence of interaction should not be interpreted as endpoint-specific equivalence.

Individual studies provide supportive surrogate evidence. In FIT, 297 women with T2DM were identified; after 3 years, alendronate increased lumbar-spine aBMD by 6.6% and hip aBMD by 2.4%, with treatment-related aBMD responses similar to those in women without diabetes, but the subgroup was not powered for fracture efficacy (75). In the pooled Japanese risedronate analysis, 53 participants with diabetes and 832 without diabetes received 48 weeks of risedronate; aBMD and BTM responses did not significantly differ by diabetes status, although fracture outcomes were not the focus (76). Together, these data support class-level skeletal responsiveness while leaving individual fracture endpoints unevenly precise.

### 7.3 Denosumab: vertebral benefit with an unresolved nonvertebral subgroup signal

The diabetes-specific denosumab evidence is more heterogeneous. In FREEDOM, 508 women with diabetes were included (denosumab n=266; placebo n=242) and followed for 3 years (77). New vertebral fractures occurred in 1.6% with denosumab versus 8.0% with placebo (RR 0.20, 95% CI 0.07–0.61; p=0.001), whereas nonvertebral fractures occurred in 11.7% versus 5.9% (HR 1.94, 95% CI 1.00–3.77; p=0.046). In the cross-trial individual-participant analysis, the denosumab nonvertebral outcome showed a significant treatment-by-diabetes interaction (p=0.01) (14). The subgroup signal is based on relatively few events and does not establish diabetes-specific harm, but it precludes a simple statement that all denosumab fracture effects are “preserved” in T2DM.

Real-world comparative studies provide complementary but nonrandomized evidence. In a Danish registry cohort of 8, 745 people with diabetes (98.2% T2DM), the adjusted hazard ratio for major osteoporotic fracture with denosumab versus alendronate was 0.89 (95% CI 0.78–1.02) (78). In a 2025 propensity-matched T2DM cohort of 415 zoledronic-acid/denosumab pairs, 5-year cumulative fracture incidence was 38% versus 31%, with an overall fracture HR of 1.17 (95% CI 0.78–1.75) for zoledronic acid versus denosumab (79). These observational estimates do not resolve the randomized FREEDOM subgroup signal because residual confounding and treatment selection remain possible.

### 7.4 General-osteoporosis safety evidence: prolonged antiresorptive suppression

The evidence used to discuss prolonged antiresorptive suppression is derived mainly from the general osteoporosis population rather than T2DM-specific trials. Long-duration bisphosphonate therapy is associated with increased atypical femoral-fracture risk, although the absolute number of typical osteoporotic fractures prevented remains substantially larger in appropriately selected high-risk patients (80). Long-term denosumab bone biopsies in postmenopausal osteoporosis show sustained very low remodeling with preserved histology (81). These findings provide safety context but cannot define a diabetes-specific “oversuppression” state.

No validated diabetes-specific definition of “oversuppression” exists, and no β-CTX-I or other BTM threshold has been shown to identify harm from antiresorptive therapy in T2DM. Accordingly, concern about excessive suppression should be treated as a monitoring issue and mechanistic hypothesis, not as a T2DM-specific contraindication to otherwise indicated therapy.

### 7.5 General-osteoporosis evidence: denosumab discontinuation and transition

Denosumab discontinuation is clinically important, but the rebound evidence cited here is not diabetes-specific. In *post hoc* analyses of FREEDOM and its Extension, withdrawal was followed by rapid BTM increases, loss of aBMD, and increased risk of multiple vertebral fractures, particularly in patients with prior vertebral fracture (82); additional analyses indicate greater multiple-vertebral-fracture risk after longer denosumab exposure (83). These data establish a general treatment-transition principle, not a validated feature of the proposed T2DM four-axis framework.

The mechanistic lesson is limited: pharmacologically suppressed remodeling can rebound after withdrawal, demonstrating that a treatment-induced turnover state is dynamic. This observation should not be used to infer the endogenous osteoclast phenotype of T2DM.

Clinically, denosumab should not be stopped or delayed without a plan for subsequent antiresorptive therapy, consistent with contemporary osteoporosis guidance (84, 85). This recommendation is based primarily on general osteoporosis evidence and should be distinguished from T2DM-specific efficacy data.

### 7.6 Clinical inference: treatment response does not validate an osteoclast mechanism

Taken together, the available data show that lower baseline BTMs in T2DM do not by themselves identify lack of response to antiresorptive therapy. The strongest evidence is class-level and pooled; agent-specific fracture precision varies, and a nonsignificant interaction is not proof of equivalence. These clinical findings support fracture-risk–based treatment but do not validate a particular endogenous osteoclast mechanism.

## 8 Treatment implications: established principles versus T2DM-specific evidence gaps

### 8.1 Current treatment principles are fracture-risk based, not turnover-threshold based

Lower formation markers in T2DM provide biological rationale for considering osteoanabolic therapy, but current treatment selection remains based on fracture risk rather than on the proposed four-axis framework. The ADA 2026 Standards recognize skeletal fragility in diabetes and retain fracture-risk–based treatment principles, while general osteoporosis guidelines support osteoanabolic or romosozumab therapy for selected very-high-risk patients followed by antiresorptive consolidation (84–86). These sequencing principles are established in general osteoporosis care; they have not been prospectively validated as a T2DM-specific phenotype-guided algorithm.

No trial has shown that a low β-CTX-I or P1NP value identifies a person with T2DM who will sustain fewer fractures with anabolic-first rather than antiresorptive-first treatment. Low BTMs therefore provide biological context but are not a current basis for choosing between these treatment classes.

### 8.2 Teriparatide and abaloparatide: surrogate skeletal responses are demonstrable; fracture evidence is limited

Teriparatide evidence in T2DM is supportive but not fracture-powered. In the DANCE observational study, 291 of 4, 042 teriparatide-treated participants had T2DM and received treatment for up to 24 months (87). In T2DM, nonvertebral fracture incidence was 3.5 per 100 patient-years during months 0–6 and 1.6 per 100 patient-years thereafter (rate ratio 0.47, 95% CI 0.12–1.87); diabetes was not a significant determinant of fracture outcome, and the treatment-duration effect did not differ by diabetes status (interaction p=0.792). Because DANCE was uncontrolled and observational, these data support treatment responsiveness rather than comparative fracture efficacy.

The 2026 T2DM-specific pilot trial randomized 129 postmenopausal women in a 1:1:1:1 ratio to zoledronate, denosumab, teriparatide, or standard care for 72 weeks; the published prespecified analysis reports only 24-week exploratory HR-pQCT and BTM outcomes (88). Teriparatide improved several trabecular vBMD and microarchitectural measures, while fracture outcomes were neither the interim endpoint nor adequately powered. The study therefore provides direct T2DM comparative surrogate evidence, not proof of fracture superiority.

In the ACTIVE *post hoc* T2DM analysis, 198 women were included (abaloparatide n=65, placebo n=65, teriparatide n=68) for 18 months (89). At month 18, the abaloparatide-versus-placebo differences in aBMD change were 7.6% (95% CI 5.9–9.3) at the lumbar spine, 3.3% (2.4–4.3) at the total hip, and 2.8% (1.7–4.0) at the femoral neck; TBS also improved. New vertebral fractures occurred in 0 abaloparatide-treated versus 3 placebo-treated participants, and other fracture counts were small. These data establish surrogate responsiveness in the T2DM subgroup, while the small *post hoc* fracture-event counts do not establish diabetes-specific antifracture superiority.

### 8.3 Romosozumab: T2DM surrogate response versus general-population fracture and safety evidence

Romosozumab has fracture-efficacy and cardiovascular-safety data from the general osteoporosis population, not from T2DM-specific fracture-powered trials. FRAME demonstrated vertebral and clinical fracture reduction versus placebo without a cardiovascular imbalance, whereas ARCH demonstrated greater fracture reduction with romosozumab followed by alendronate than with alendronate alone but showed a numerical excess of serious cardiovascular events during the first year (90, 91). These trials inform current safety and sequencing principles but should not be presented as diabetes-specific outcome evidence.

The diabetes subgroup analysis of ARCH addressed surrogate skeletal outcomes. It included 360 women with diabetes (357 with T2DM; romosozumab-to-alendronate n=165 and alendronate-to-alendronate n=195) followed for 36 months (92). At month 12, the between-group least-squares mean difference favored romosozumab by 7.0% for lumbar-spine aBMD (p<0.001) and 2.6% for tissue-thickness–adjusted TBS (p<0.001); the differences persisted during subsequent alendronate therapy. This supports aBMD/TBS responsiveness in a predominantly T2DM subgroup, but the analysis was not designed to establish diabetes-specific fracture superiority.

A 2026 single-center real-world cohort provides a counterpoint. Among 87 romosozumab-treated patients, 34 had diabetes; after 12 months, median aBMD gains were smaller in those with diabetes than in those without diabetes at the lumbar spine (3.1% vs 9.9%; p=0.020), total hip (0.3% vs 4.1%; p=0.027), and femoral neck (0.1% vs 3.9%; p=0.028) (93). The retrospective design, small diabetic subgroup, differences in age and BMI, and mixed diabetes characterization preclude causal inference, but the study argues against assuming uniform anabolic responsiveness.

Because T2DM itself is associated with elevated cardiovascular risk, romosozumab decisions should follow current guideline-based cardiovascular risk assessment. The FRAME/ARCH cardiovascular observations are general-population evidence; they do not justify a diabetes-specific recommendation beyond existing guidance (85, 90, 91).

### 8.4 Treatment sequencing: established osteoporosis principle, unvalidated T2DM phenotype algorithm

Osteoanabolic therapy is generally followed by antiresorptive treatment to preserve skeletal gains, and denosumab discontinuation requires planned transition to another antiresorptive agent (82–86). These are established osteoporosis-management principles. They have not been prospectively validated through the proposed four-axis T2DM framework, and the framework should not be interpreted as a current sequencing algorithm.

In T2DM, treatment history, adherence, renal and cardiovascular status, prior fracture pattern, and ability to complete a planned sequence remain clinically relevant. Baseline β-CTX-I or P1NP should not be used as a stand-alone selector of anabolic versus antiresorptive therapy.

### 8.5 Can β-CTX-I and P1NP become treatment-selection biomarkers?

At present, no. P1NP and β-CTX-I are standardized reference BTMs for assessing remodeling and monitoring therapy (17), but there is no prospectively validated T2DM threshold that selects anabolic over antiresorptive therapy. Furthermore, BTMs do not predict incident fracture in T2DM as reliably as they do in people without diabetes (94).

A clinically useful biomarker must demonstrate predictive interaction, not simply association. A future trial would need to stratify patients by predefined turnover phenotype, randomize within each stratum to alternative treatment sequences, and show that baseline turnover modifies fracture benefit. Until then, β-CTX-I and P1NP should provide biological context rather than dictate a prescription.

Quantitative treatment estimates and their evidence provenance are summarized in **Table 2**; surrogate outcomes are separated from fracture endpoints wherever the source data permit.

**Table 2:** Quantitative clinical evidence for osteoporosis pharmacotherapy in type 2 diabetes mellitus and evidence provenance.

Study/evidence provenance	T2DM sample/follow-up	Quantitative outcome(s)	Effect modification/comparator	Interpretation/limitation
Pooled antiresorptives Eastell et al., 2022 (14) T2DM subgroup across 15 RCTs	Nonvertebral/all/hip: n=6, 539; vertebral: n=3, 787. BMD analysis: n=2, 266–3, 124; BTM: n=246–447. BMD at 24 mo; BTM at 3–12 mo.	Fracture in T2DM: vertebral OR 0.48 (95% CI 0.36–0.63; 215/3, 787); nonvertebral HR 0.86 (0.74–1.00; 666/6, 539); all HR 0.74 (0.64–0.84; 880/6, 539); hip HR 0.82 (0.57–1.16; 125/6, 539). Total-hip aBMD active−placebo: +3.86% (3.54–4.18). CTX: −53.2% (−59.5 to −45.9).	Interaction p: vertebral 0.53; nonvertebral 0.58; all 0.80; hip 0.33; total-hip aBMD 0.95; CTX 0.69.	No statistically detectable pooled effect modification by T2DM. Does not establish formal equivalence; drug-class and endpoint precision vary.
Bisphosphonate subset Eastell et al., 2022 (14) 9 RCTs	Nonvertebral/all/hip: n=1, 476; vertebral: n=1, 060. BMD: n=385–791; BTM: n=119–265.	Vertebral OR 0.38 (0.23–0.64; 74/1, 060); nonvertebral HR 0.77 (0.56–1.06; 152/1, 476); all HR 0.64 (0.49–0.83; 217/1, 476); hip HR 1.13 (0.58–2.20; 37/1, 476). Total-hip aBMD active−placebo: +4.08% (3.42–4.75); CTX: −51.1% (−59.8 to −40.5).	Interaction p: vertebral 0.21; nonvertebral 0.51; all 0.32; hip 0.14; total-hip aBMD 0.20; CTX 0.88.	Most consistent class-specific evidence. Hip estimate is imprecise; no-interaction is not proof of equivalence.
Alendronate Keegan et al., 2004 (75) FIT diabetes subgroup	T2DM n=297; 3 y.	Lumbar-spine aBMD +6.6%; hip aBMD +2.4% with alendronate over 3 y. Placebo groups lost aBMD at most sites.	aBMD response to alendronate vs placebo was similar in women with and without diabetes.	Supports surrogate responsiveness; not powered for T2DM-specific fracture efficacy.
Risedronate Inoue et al., 2016 (76) *Post hoc* pooled phase III data	DM n=53; non-DM n=832; 48 wk.	Across all 885 risedronate-treated participants, BMD + 5.52%, uNTX −35.4%, BAP −33.8%; treatment responses did not significantly differ by DM status.	DM vs non-DM response differences not statistically significant.	Surrogate outcomes only; small DM subgroup; pooled percentage changes are not DM-specific estimates.
Denosumab Ferrari et al., 2020 (77) FREEDOM diabetes subgroup	Diabetes n=508: denosumab 266, placebo 242; 3 y randomized phase.	New vertebral fracture: 1.6% vs 8.0%, RR 0.20 (0.07–0.61), p=0.001. Nonvertebral fracture: 11.7% vs 5.9%, HR 1.94 (1.00–3.77), p=0.046. aBMD increased with denosumab.	Cross-trial IPD analysis: significant treatment×diabetes interaction for nonvertebral fracture (p=0.01).	Vertebral benefit and aBMD response demonstrated; nonvertebral signal unresolved and event-limited.
Denosumab vs alendronate Viggers et al., 2022 (78) Nationwide observational cohort	Diabetes n=8, 745 (98.2% T2DM): alendronate 8, 255; denosumab 490; registry follow-up to 2018.	Adjusted HR for major osteoporotic fracture with denosumab vs alendronate: 0.89 (95% CI 0.78–1.02).	Head-to-head observational comparator.	No clear difference detected; substantial baseline treatment-channeling differences and residual confounding remain.
Zoledronic acid vs denosumab Rouach et al., 2025 (79) Propensity-matched T2DM cohort	415 matched pairs; 5-y cumulative outcomes reported.	5-y cumulative fracture incidence: 38% (zoledronic acid) vs 31% (denosumab). Overall fracture HR 1.17 (0.78–1.75).	Head-to-head observational comparator.	No statistically significant difference; observational design cannot resolve randomized subgroup signals.
Teriparatide Schwartz et al., 2016 (87) DANCE observational analysis	T2DM n=291 of 4, 042; treatment up to 24 mo, then observation up to 24 mo.	Nonvertebral fracture incidence in T2DM: 3.5/100 patient-y at 0–6 mo vs 1.6/100 patient-y thereafter; rate ratio 0.47 (95% CI 0.12–1.87). Spine/hip aBMD increased.	Duration×diabetes interaction p=0.792; diabetes not a significant fracture determinant (p=0.858).	Uncontrolled observational study; supports responsiveness, not comparative fracture efficacy.
Zoledronate/denosumab/teriparatide Prasad et al., 2026 (88) T2DM-specific randomized pilot	n=129; 1:1:1:1 allocation; planned 72 wk; published prespecified 24-wk interim analysis.	Teriparatide improved several trabecular vBMD/microarchitectural measures; denosumab improved selected tibial measures; zoledronate improved tibial total vBMD. BTMs changed in expected directions.	No fracture-powered comparison in the published interim analysis.	Direct T2DM comparative evidence, but exploratory surrogate endpoints and small single-center sample.
Abaloparatide Dhaliwal et al., 2020 (89) ACTIVE T2DM *post hoc* subgroup	n=198: abaloparatide 65, placebo 65, teriparatide 68; 18 mo.	Abaloparatide−placebo aBMD difference at 18 mo: lumbar spine +7.6% (5.9–9.3); total hip +3.3% (2.4–4.3); femoral neck +2.8% (1.7–4.0). New vertebral fractures: 0 vs 3; other fracture counts small.	*Post hoc* subgroup; no adequately powered diabetes-specific treatment interaction for fracture endpoints.	Strong surrogate response; fracture evidence too sparse for diabetes-specific superiority.
Romosozumab→alendronate vs alendronate Ferrari et al., 2025 (92) ARCH diabetes *post hoc* analysis	Diabetes n=360 (357 T2DM; 3 T1D): 165 romosozumab→alendronate, 195 alendronate; 36 mo.	Month-12 between-group difference: lumbar-spine aBMD +7.0% (p<0.001); tissue-thickness–adjusted TBS + 2.6% (p<0.001). Differences persisted at months 24/36.	Comparator analysis of surrogate endpoints; not fracture-powered for T2DM.	Supports predominantly T2DM aBMD/TBS responsiveness; does not establish diabetes-specific fracture superiority.
Romosozumab Alshamsi et al., 2026 (93) Single-center real-world cohort	n=87; diabetes n=34, no diabetes n=53; 12 mo.	Median aBMD gain, diabetes vs no diabetes: lumbar spine 3.1% vs 9.9% (p=0.020); total hip 0.3% vs 4.1% (p=0.027); femoral neck 0.1% vs 3.9% (p=0.028).	Observational diabetes-status comparison.	Suggests heterogeneous response; small, retrospective, older/higher-BMI diabetic subgroup; diabetes type not fully restricted to T2DM.

aBMD, areal bone mineral density; BTM, bone turnover marker; CI, confidence interval; CTX, C-terminal telopeptide as reported by source studies; HR, hazard ratio; HR-pQCT, high-resolution peripheral quantitative computed tomography; OR, odds ratio; RCT, randomized controlled trial; TBS, trabecular bone score; T2DM, type 2 diabetes mellitus. “No significant interaction” indicates that effect modification by diabetes was not statistically detected; it does not establish treatment equivalence. General-osteoporosis safety or sequencing evidence is identified explicitly in the text and is not presented as T2DM-specific efficacy evidence.

### 8.6 Emerging osteoclast-targeted strategies: evidence stage and translational boundaries

The translational value of the framework lies in separating established osteoporosis targets from T2DM-specific hypotheses. RANKL–RANK inhibition is clinically established in osteoporosis and has diabetes-subgroup outcome data, but this does not make RANKL a T2DM-specific therapeutic target. By contrast, ATP6V0D2/DC-STAMP-dependent fusion, RAGE-dependent cytoskeletal organization, AGE-modified matrix/resorption interfaces, and osteoclast-derived coupling signals remain experimental or mechanistic research directions rather than validated interventions for diabetic bone fragility (29, 34, 35, 73).

Accordingly, next-generation development should prioritize testable questions rather than assume target readiness: whether stage-selective modulation can alter fusion or mature resorption without eliminating the lineage; whether matrix-aware approaches can distinguish cellular suppression from substrate resistance; whether pathological resorption can be reduced while preserving formation-coupling signals; and whether biomarker-enriched trials can identify remodeling phenotypes that predict differential treatment benefit. None of these strategies currently provides a T2DM treatment recommendation. Their evidence stage and translational boundaries are summarized in **Table 3**.

**Table 3:** Osteoclast-targeted drug-discovery directions, evidence stage, and translational boundaries relevant to T2DM.

Strategy/evidence anchor	Biological stage	Direct relevance to T2DM	Development stage	Translational boundary
RANKL–RANK inhibition (14, 77)	Commitment and differentiation	Clinical pooled/subgroup evidence shows treatment response in people with T2DM; this establishes clinical relevance but not a T2DM-specific target mechanism.	Established osteoporosis target; T2DM subgroup evidence available	Agent-specific fracture precision, rebound after denosumab withdrawal, and coupling effects require separate interpretation.
ATP6V0D2/DC-STAMP modulation (29)	Fusion and multinucleation	Support derives from a high-glucose RAW264.7 model showing reduced fusion-related gene expression; no direct human T2DM target validation is available.	Preclinical mechanistic	Human relevance, reversibility, dose window, and skeletal selectivity are unknown.
RAGE/cytoskeletal organization (34)	Polarization and sealing-zone function	RAGE regulates osteoclast cytoskeletal organization in genetic/preclinical models, but the cited evidence is not a T2DM therapeutic-validation study.	Preclinical mechanistic	RAGE is pleiotropic and systemic modulation may have substantial off-target effects.
AGE-modified matrix/resorption interface (35)	Mature matrix degradation	AGE-modified mineralized matrix reduces osteoclast-mediated collagen degradation experimentally, supporting substrate resistance as a mechanistic hypothesis.	Experimental mechanistic	No intervention has established this interface as a therapeutic target in human T2DM; *in vivo* contribution to circulating CTX and fracture risk is unquantified.
Coupling-preserving modulation (73)	Reversal and formation coupling	Human bone-biopsy studies identify osteoclast-derived coupling factors, but these data were not generated as T2DM-specific target-validation studies.	Human mechanistic; hypothesis-generating	The relevant coupling factors, therapeutic window, and response biomarkers in T2DM remain undefined.
Biomarker-enriched trial design (17, 94)	Patient selection rather than an osteoclast target	β-CTX-I and P1NP are reference turnover markers, but no prospectively validated T2DM threshold predicts preferential response to anabolic or antiresorptive therapy.	Clinical research strategy; not a drug target	Requires prespecified biomarker strata and demonstration of treatment-by-biomarker interaction on clinically meaningful outcomes.

AGE, advanced glycation end product; BTM, bone turnover marker; CTX, C-terminal telopeptide of type I collagen as reported by source studies; P1NP, procollagen type I N-terminal propeptide; RAGE, receptor for advanced glycation end products; RANKL, receptor activator of nuclear factor-κB ligand; T2DM, type 2 diabetes mellitus; β-CTX-I, β-isomerized C-terminal telopeptide of type I collagen.

### 8.7 Toward phenotype-informed therapy

A future phenotype-informed model should combine established fracture risk with five diabetes-relevant domains: fracture history and skeletal severity; aBMD/TBS and turnover context; renal and metabolic phenotype; falls, neuropathy, and sarcopenia; and cardiovascular risk, prior osteoporosis therapy, and adherence. Longer diabetes duration remains relevant because it increases fracture risk beyond standard FRAX estimates (65).

In such a framework, low turnover would not independently trigger anabolic therapy. Rather, it might help distinguish a patient whose fragility is dominated by impaired tissue renewal from one whose clinical risk is adequately addressed by standard antiresorptive therapy. This remains a research framework, not a validated treatment algorithm.

Current pharmacological evidence is summarized in **Table 2**, emerging osteoclast-targeted research directions are summarized in **Table 3**, and the separation between current care and unvalidated research hypotheses is illustrated in **Figure 3**.

**Figure 3:**
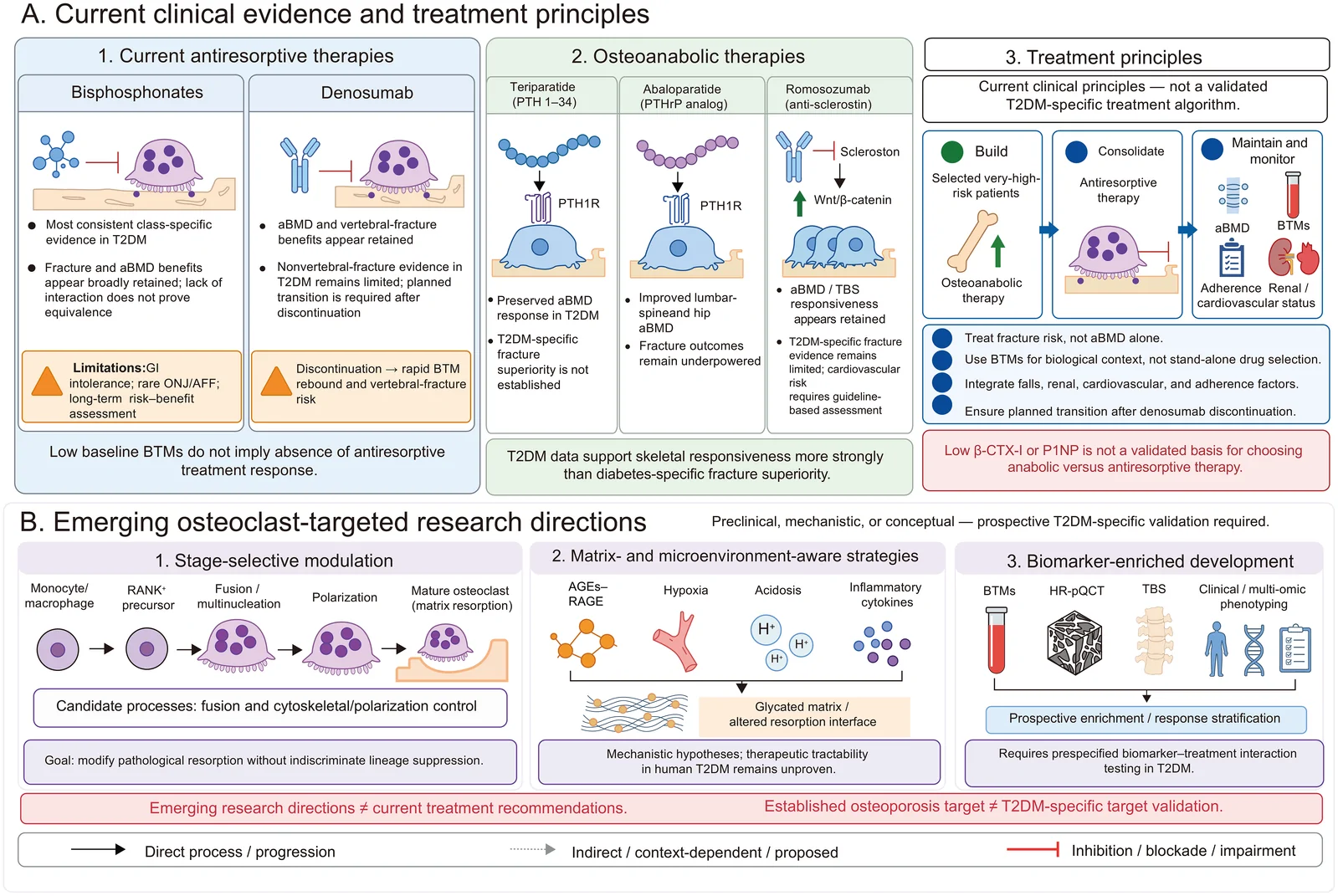
Therapeutic evidence and research directions in T2DM bone fragility. Panel **(A)** separates current clinical evidence and treatment principles from unvalidated T2DM-specific treatment algorithms. Pooled antiresorptive analyses have not detected significant treatment-by-diabetes interactions for major fracture outcomes, but this does not establish formal equivalence; denosumab has diabetes-subgroup vertebral-fracture benefit with an unresolved nonvertebral signal. Osteoanabolic studies in T2DM support aBMD, TBS, or other surrogate skeletal responsiveness more strongly than diabetes-specific fracture prevention. Current care remains fracture-risk based, and low β-CTX-I or P1NP is not a validated treatment-selection threshold. Panel **(B)** summarizes emerging osteoclast-targeted research directions—stage-selective modulation, matrix- and microenvironment-aware strategies, and biomarker-enriched development. These are preclinical, mechanistic, or conceptual directions that require prospective T2DM-specific validation, not current treatment recommendations. An established osteoporosis target should not be interpreted as a T2DM-specific validated target.

## 9 Discussion and research agenda: from the osteoclast paradox to actionable skeletal phenotypes

### 9.1 Limitations of the current evidence and framework

The osteoclast paradox is a heuristic framework rather than a validated clinical phenotype. Its principal limitation is that the constituent observations are drawn from different evidence streams. No current human study has simultaneously established a low circulating-BTM phenotype, an altered osteoclast-lineage state, tissue-level remodeling differences, matrix abnormalities, and treatment response in the same T2DM participants. Direct human osteoclast-lineage evidence is particularly sparse: the precursor phenotype highlighted in this Review derives largely from one small postmenopausal cohort, while histomorphometric evidence is also based on limited tissue samples. Experimental studies establish mechanistic plausibility but cannot be assumed to reproduce the metabolic context of human T2DM.

A second limitation is confounding by overlapping metabolic and treatment exposures. Obesity and body composition, mechanical loading, insulin resistance, renal and microvascular disease, glucose-lowering or weight-loss therapy, hypoglycemia, and prior osteoporosis treatment are variably measured across studies. Some observations attributed to T2DM may therefore partly reflect these coexisting exposures or DXA soft-tissue effects rather than diabetes-specific osteoclast biology. Future studies should separate and model these dimensions explicitly.

Circulating BTMs are whole-skeleton biochemical outputs influenced by preanalytic conditions, renal function, assay methodology, and matrix degradation; they are not direct readouts of osteoclast number, mature resorptive function, or BMU activation and kinetics. Treatment evidence answers a different question again—whether therapies retain surrogate or clinical effects—not whether endogenous mature osteoclast resorptive function is increased or preserved. Consequently, antiresorptive treatment response should not be treated as mechanistic validation of the four-axis model. Treatment evidence is also uneven, with many agent-specific conclusions derived from *post hoc* subgroups or observational cohorts and no fracture-powered T2DM trials comparing anabolic-first with antiresorptive-first strategies. Evidence-type labels in the tables are descriptive categories intended to clarify provenance and directness; they are not formal GRADE ratings or substitutes for study-level risk-of-bias assessment. Because this is a narrative review without formal risk-of-bias grading, the framework should be considered hypothesis-generating; conclusions are strongest where replicated human data converge and weakest where inference depends on isolated cellular, tissue, or preclinical studies.

### 9.2 Defining human osteoclast states and matrix interactions

The first priority is to replicate the precursor–function dissociation in larger, diverse cohorts using precursor phenotyping, tartrate-resistant acid phosphatase isoform 5b (TRACP5b), β-CTX-I, ex vivo fusion and pit resorption, and metabolic characterization in the same participants (12). Stage-resolved single-cell, metabolic, cytoskeletal, and functional assays should determine whether the dominant bottleneck lies in commitment, fusion, polarization, acidification, proteolysis, survival, or coupling.

The glycated-matrix hypothesis also requires direct human testing. Standardized osteoclast populations should be cultured on diabetic and nondiabetic human bone while AGE chemistry, pit formation, acidification, collagen-fragment release, and cell survival are measured (35). This design would separate cellular suppression from substrate resistance.

### 9.3 Resolving spatial and metabolic skeletal phenotypes

Systemic BTMs should be integrated with HR-pQCT, site-specific imaging, and—where feasible—bone tissue to determine whether focal remodeling states coexist with low whole-skeleton biochemical output. Prospective phenotyping should additionally incorporate adiposity and body composition, insulin sensitivity, β-cell function, glycemic variability, PTH/renal status, microvascular disease, medication and weight-loss exposure, complications, falls, aBMD, TBS, and prior osteoporosis treatment. Analyses should test whether putative T2DM-specific skeletal features persist after accounting for these overlapping exposures. Statistical clusters are clinically meaningful only if they are reproducible and predict fracture or differential treatment response.

### 9.4 Predictive biomarkers and treatment-response trials

To establish predictive utility, biomarker-stratified randomized trials should test treatment-by-biomarker interactions with fracture endpoints and compare complete sequences: anabolic followed by antiresorptive, antiresorptive followed by anabolic, and potent antiresorptive therapy alone. Such trials are required before P1NP or β-CTX-I can move from monitoring tools to validated treatment-selection biomarkers in T2DM (17, 94).

Treatment discontinuation and contemporary diabetes therapy must be incorporated. Denosumab rebound is established generally, but whether diabetic remodeling modifies its magnitude is unknown (82, 83). GLP-1 receptor agonists, SGLT2 inhibitors, thiazolidinediones, pharmacological weight loss, lean-mass change, hypoglycemia, and falls can all alter the skeletal context and should be prospectively documented (49–55).

### 9.5 Drug discovery and translational roadmap

Translation should proceed through three levels: Level 1, define the abnormal osteoclast/BMU state; Level 2, identify that state reproducibly in patients using biomarkers, imaging, and metabolic variables; and Level 3, demonstrate that the phenotype predicts differential fracture benefit in prospective trials. The recent maltol study provides a proof-of-concept example of how the diabetic milieu may amplify an external metabolite, but a single unreplicated mechanism should not be treated as a dominant driver (95).

Only after prospective Level 3 validation should a remodeling phenotype become a clinical decision rule. Priority questions and preferred designs are summarized in **Table 4**, while candidate osteoclast-targeted drug-discovery strategies are summarized in **Table 3**.

**Table 4:** Priority research gaps for phenotype-informed management of diabetic bone fragility.

Priority question	Current limitation	Preferred study design/measurements	Potential clinical impact
Human osteoclast state and life-cycle bottleneck	Direct human osteoclast evidence is sparse and small; most experiments measure a single differentiation endpoint.	Large multimodal human cohorts combining precursor phenotyping, TRACP5b/β-CTX-I, ex vivo fusion and pit resorption, plus stage-resolved single-cell, metabolic, cytoskeletal, acidification, and resorption assays.	Validate reproducible human osteoclast phenotypes and identify biologically druggable stages.
Glycated-matrix degradability	Experimental substrate evidence exists (35), but the *in vivo* contribution of matrix resistance to low circulating CTX/β-CTX-I is unknown.	Standardized osteoclast populations on diabetic vs nondiabetic human bone with AGE chemistry, acidification, pit formation, and collagen-fragment release.	Separate cellular suppression from substrate resistance.
Spatial and metabolic skeletal phenotypes	Systemic BTMs average the skeleton, while T2DM is often treated as one exposure despite marked metabolic heterogeneity.	BTMs + HR-pQCT/site-specific imaging ± tissue sampling integrated with insulin sensitivity, glycemic variability, renal/PTH status, microvascular disease, and fractures.	Define reproducible phenotypes that capture focal remodeling and metabolic context.
Predictive biomarkers	P1NP and β-CTX-I are validated for turnover/monitoring, not treatment selection in T2DM (17, 94).	Biomarker-stratified randomized trials with prespecified treatment-by-biomarker interactions and fracture endpoints.	Determine whether turnover biology can guide therapy rather than merely describe it.
Anabolic-first and treatment sequencing	Direct T2DM trials are small and predominantly surrogate-based (88).	Multicenter fracture-powered trials comparing complete sequences: anabolic → antiresorptive, antiresorptive → anabolic, and potent antiresorptive therapy alone.	Test whether specific T2DM phenotypes preferentially benefit from anabolic-first treatment.
Denosumab discontinuation/transition	Rebound biology is established generally (82, 83), but modification by diabetic remodeling is unknown.	Prospective transition studies with BTMs, aBMD, adherence, treatment duration, and vertebral-fracture outcomes.	Optimize safe long-term sequencing and discontinuation management.

aBMD, areal bone mineral density; BTM, bone turnover marker; CTX, C-terminal telopeptide of type I collagen as reported by source studies; HR-pQCT, high-resolution peripheral quantitative computed tomography; P1NP, procollagen type I N-terminal propeptide; PTH, parathyroid hormone; T2DM, type 2 diabetes mellitus; TRACP5b, tartrate-resistant acid phosphatase isoform 5b; β-CTX-I, β-isomerized C-terminal telopeptide of type I collagen.

## 10 Conclusions

T2DM is associated with increased skeletal fragility together with a population-level pattern of lower circulating bone-turnover markers and frequently non-low DXA-derived aBMD (9–11). These observations are clinically important but arise from distinct measurement domains and do not, by themselves, establish a single osteoclast mechanism.

In this Review, the term “osteoclast paradox” is therefore used as an author-proposed heuristic for organizing apparent dissociations among circulating BTMs, osteoclast-lineage state, mature resorptive function, BMU/tissue remodeling, matrix properties, and treatment response. Direct human evidence connecting these levels within the same individuals remains limited, and the four-axis framework should be regarded as a testable research model rather than a validated clinical phenotype.

Available treatment evidence indicates that low baseline BTM concentrations do not by themselves establish absence of response to antiresorptive therapy, but treatment response is not mechanistic proof of preserved endogenous mature osteoclast resorptive function. Pooled antiresorptive analyses have not detected statistically significant treatment-by-diabetes interactions for major fracture outcomes, yet this does not prove formal equivalence and agent-specific precision varies. Osteoanabolic evidence in T2DM currently supports aBMD, TBS, and early microarchitectural responsiveness more strongly than diabetes-specific fracture prevention. Current treatment should therefore remain fracture risk–based rather than turnover-threshold based. Future progress will require paired human studies that measure biomarkers, osteoclast-lineage state, mature functional competence, tissue or imaging phenotypes, and treatment outcomes in the same well-characterized T2DM populations.
