# Supplementary material for: The osteoclast paradox in type 2 diabetes: low turnover, skeletal fragility, and implications for osteoclast-targeted therapy
Source: Front Endocrinol (Lausanne). 2026 Oct 30;17:1972283. doi: 10.3389/fendo.2026.1972283 (PMC13760506; doi:10.3389/fendo.2026.1972283)
Supplement: Supplementary file 1 [file Table1.docx]

**Supplementary Table S1**

**The Osteoclast Paradox in Type 2 Diabetes: Low Turnover, Skeletal Fragility, and Implications for Osteoclast-Targeted Therapy**

*Literature-search strategy, study selection, evidence appraisal, and source verification*

This review was designed as a mechanism-oriented narrative review rather than a systematic review. PubMed/MEDLINE was searched from database inception to 28 July 2026, supplemented by backward and forward citation tracking and targeted searches of journal and publisher websites. The original search was iterative; exact historical retrieval counts and duplicate-screening logs were not prospectively retained. Therefore, no PRISMA flow diagram or retrospective pseudo-precise screening counts were generated. For transparency and reproducibility, the prespecified concept blocks used during the review were translated during revision into the explicit PubMed syntax below.

| **Search block** | **Concept** | **Explicit PubMed/MEDLINE syntax used for reproducibility in revision** | **Purpose** |
| --- | --- | --- | --- |
| S1-1 | T2DM skeletal phenotype, fracture, aBMD and turnover markers | ("Diabetes Mellitus, Type 2"[MeSH Terms] OR "type 2 diabetes"[Title/Abstract] OR T2DM[Title/Abstract]) AND (bone[Title/Abstract] OR skeletal[Title/Abstract] OR osteoporosis[Title/Abstract] OR fracture*[Title/Abstract]) AND ("bone turnover"[Title/Abstract] OR P1NP[Title/Abstract] OR CTX[Title/Abstract] OR osteocalcin[Title/Abstract] OR "bone mineral density"[Title/Abstract] OR BMD[Title/Abstract]) | Population-level phenotype, fracture risk, and biochemical remodeling output. |
| S1-2 | Osteoclast lineage, differentiation and mature function | ("type 2 diabetes"[Title/Abstract] OR T2DM[Title/Abstract] OR hyperglyc*[Title/Abstract]) AND (osteoclast*[Title/Abstract] OR RANKL[Title/Abstract] OR RANK[Title/Abstract] OR NFATc1[Title/Abstract] OR TRAP[Title/Abstract] OR TRACP[Title/Abstract]) AND (precursor*[Title/Abstract] OR differentiation[Title/Abstract] OR fusion[Title/Abstract] OR multinucleation[Title/Abstract] OR polarization[Title/Abstract] OR resorption[Title/Abstract] OR "pit formation"[Title/Abstract]) | Lineage abundance, differentiation stage, fusion/polarization, and mature resorptive competence. |
| S1-3 | Bone histomorphometry and BMU/tissue remodeling | ("type 2 diabetes"[Title/Abstract] OR T2DM[Title/Abstract] OR diabetes[Title/Abstract]) AND (histomorphometr*[Title/Abstract] OR "bone biopsy"[Title/Abstract] OR "bone remodeling"[Title/Abstract] OR "basic multicellular unit"[Title/Abstract] OR BMU[Title/Abstract]) | Tissue-level turnover, formation/resorption indices, reversal phase, and coupling. |
| S1-4 | AGEs, RAGE, glycation and matrix degradability | ("type 2 diabetes"[Title/Abstract] OR T2DM[Title/Abstract] OR diabetes[Title/Abstract]) AND ("advanced glycation end products"[Title/Abstract] OR AGE[Title/Abstract] OR AGEs[Title/Abstract] OR RAGE[Title/Abstract] OR glycat*[Title/Abstract] OR "collagen cross-link*"[Title/Abstract]) AND (bone[Title/Abstract] OR osteoclast*[Title/Abstract] OR matrix[Title/Abstract] OR resorption[Title/Abstract]) | Cell-intrinsic AGE/RAGE effects and substrate-level effects of glycated matrix. |
| S1-5 | Bone quality, microarchitecture and osteocyte/BMU biology | ("type 2 diabetes"[Title/Abstract] OR T2DM[Title/Abstract]) AND ("bone quality"[Title/Abstract] OR microarchitect*[Title/Abstract] OR "HR-pQCT"[Title/Abstract] OR "cortical porosity"[Title/Abstract] OR TBS[Title/Abstract] OR osteocyte*[Title/Abstract] OR sclerostin[Title/Abstract] OR microdamage[Title/Abstract] OR mechanosens*[Title/Abstract]) | Material properties, microarchitecture, osteocyte signaling, microdamage/repair, and tissue aging. |
| S1-6 | Metabolic and vascular modifiers | ("type 2 diabetes"[Title/Abstract] OR T2DM[Title/Abstract]) AND (obesity[Title/Abstract] OR adiposity[Title/Abstract] OR "insulin resistance"[Title/Abstract] OR hyperinsulinemia[Title/Abstract] OR "glycemic variability"[Title/Abstract] OR PTH[Title/Abstract] OR renal[Title/Abstract] OR microvascular[Title/Abstract] OR perfusion[Title/Abstract] OR hypoxia[Title/Abstract] OR "marrow fat"[Title/Abstract]) AND (bone[Title/Abstract] OR skeletal[Title/Abstract] OR osteoclast*[Title/Abstract] OR remodeling[Title/Abstract]) | Obesity/body composition, insulin phenotype, renal-mineral factors, marrow adiposity, perfusion and microvascular context. |
| S1-7 | Antiresorptive therapy in T2DM | ("type 2 diabetes"[Title/Abstract] OR T2DM[Title/Abstract] OR diabetes[Title/Abstract]) AND (bisphosphonate*[Title/Abstract] OR alendronate[Title/Abstract] OR risedronate[Title/Abstract] OR zoledronic[Title/Abstract] OR denosumab[Title/Abstract]) AND (fracture*[Title/Abstract] OR aBMD[Title/Abstract] OR "bone mineral density"[Title/Abstract] OR P1NP[Title/Abstract] OR CTX[Title/Abstract] OR "bone turnover"[Title/Abstract]) | T2DM-specific or diabetes-subgroup evidence for skeletal responsiveness and fracture outcomes. |
| S1-8 | Osteoanabolic therapy in T2DM | ("type 2 diabetes"[Title/Abstract] OR T2DM[Title/Abstract] OR diabetes[Title/Abstract]) AND (teriparatide[Title/Abstract] OR abaloparatide[Title/Abstract] OR romosozumab[Title/Abstract] OR osteoanabolic[Title/Abstract] OR anabolic[Title/Abstract]) AND (fracture*[Title/Abstract] OR aBMD[Title/Abstract] OR "bone mineral density"[Title/Abstract] OR TBS[Title/Abstract]) | T2DM-specific response evidence for anabolic/dual-action therapy and limits of fracture inference. |
| S1-9 | Treatment sequencing and denosumab discontinuation | ("osteoporosis"[Title/Abstract] OR "type 2 diabetes"[Title/Abstract] OR T2DM[Title/Abstract]) AND (denosumab[Title/Abstract] OR "treatment sequence*"[Title/Abstract] OR sequential[Title/Abstract] OR consolidation[Title/Abstract] OR discontinuation[Title/Abstract]) AND (fracture*[Title/Abstract] OR rebound[Title/Abstract] OR "bone turnover"[Title/Abstract] OR BMD[Title/Abstract]) | General-osteoporosis treatment principles distinguished from T2DM-specific evidence. |
| S1-10 | Glucose-lowering medications and skeletal modifiers | ("type 2 diabetes"[Title/Abstract] OR T2DM[Title/Abstract]) AND (metformin[Title/Abstract] OR insulin[Title/Abstract] OR SGLT2[Title/Abstract] OR "SGLT2 inhibitor*"[Title/Abstract] OR "GLP-1"[Title/Abstract] OR "GLP-1 receptor agonist*"[Title/Abstract] OR thiazolidinedione*[Title/Abstract] OR pioglitazone[Title/Abstract] OR "weight loss"[Title/Abstract]) AND (fracture*[Title/Abstract] OR bone[Title/Abstract] OR skeletal[Title/Abstract] OR BMD[Title/Abstract]) | Medication exposure and weight loss as modifiers/confounders of the observed skeletal phenotype. |
| S1-11 | Osteoclast-targeted drug discovery | (osteoclast*[Title/Abstract] OR RANKL[Title/Abstract] OR RANK[Title/Abstract] OR "cathepsin K"[Title/Abstract] OR "osteoclast fusion"[Title/Abstract] OR "osteoclast polarization"[Title/Abstract]) AND (target*[Title/Abstract] OR inhibitor*[Title/Abstract] OR therapeut*[Title/Abstract] OR "drug discovery"[Title/Abstract]) AND (bone[Title/Abstract] OR osteoporosis[Title/Abstract] OR diabetes[Title/Abstract]) | Emerging targets, development stage, and distinction between established osteoporosis targets and T2DM-specific validation. |

**Selection, appraisal and conflict-handling rules**

| **Item** | **Operational rule** |
| --- | --- |
| **Review type** | Focused mechanism-oriented narrative review; structured but non-systematic evidence identification. |
| **Database and date** | PubMed/MEDLINE, database inception to 28 July 2026; supplemented by backward/forward citation tracking and targeted journal/publisher website searches. |
| **Priority evidence** | Adult human T2DM cohort, case-control, cross-sectional, bone-biopsy/histomorphometric, human cellular, randomized-trial, individual-participant, systematic-review/meta-analytic, and major guideline evidence. |
| **Use of preclinical evidence** | Animal and in vitro studies were retained when they addressed mechanistic questions not directly resolvable in humans (e.g., differentiation stage, matrix degradability, cytoskeletal organization, coupling). |
| **Core exclusions** | Type 1 or gestational diabetes when used as direct evidence for the T2DM phenotype; pediatric populations; non-peer-reviewed conference abstracts; reports without a directly relevant skeletal, remodeling, osteoclast-lineage, bone-quality, fracture, or treatment endpoint. |
| **Language handling** | No database-level language filter was applied. Retained studies required sufficient English-language abstract or full-text information to permit appraisal of design, population, methods and outcomes. |
| **Multiple reports from same cohort/trial** | The most informative report for the question of interest was prioritized; companion analyses were retained when they contributed a distinct endpoint. |
| **Evidence weighting** | For population-level phenotype and treatment claims, direct human T2DM data, adequately characterized cohorts, randomized comparisons and replicated findings received greatest weight. For mechanisms, direct human tissue/cellular evidence was separated from animal/in vitro evidence, which was used mainly for biological plausibility. |
| **Additional appraisal factors** | Sample size; assay/imaging standardization; control of major confounders; directness of endpoint; independent replication; consistency with complementary evidence. |
| **Contradictory evidence** | Contradictory findings were retained and discussed rather than selectively excluded. Differences in species, insulin/glucose state, cell source, skeletal site, differentiation stage and assay were used to interpret heterogeneity. |
| **Evidence-table inclusion** | Studies were selected for evidence tables when they directly informed a central claim, represented an important counterexample, or materially affected clinical interpretation, and when sufficient methodological detail was available. |
| **General osteoporosis vs T2DM evidence** | Evidence extrapolated from the general osteoporosis population was identified separately from T2DM-specific or diabetes-subgroup evidence. |
| **Screening records** | Exact historical retrieval counts, formal duplicate-screening logs and study-level risk-of-bias grading were not prospectively recorded; no retrospective PRISMA counts were constructed. |
