# Supplementary material for: The osteoclast paradox in type 2 diabetes: low turnover, skeletal fragility, and implications for osteoclast-targeted therapy
Source: Front Endocrinol (Lausanne). 2026 Oct 30;17:1972283. doi: 10.3389/fendo.2026.1972283 (PMC13760506; doi:10.3389/fendo.2026.1972283)
Supplement: Supplementary file 2 [file Table2.docx]

**Supplementary Table S2**

**The Osteoclast Paradox in Type 2 Diabetes: Low Turnover, Skeletal Fragility, and Implications for Osteoclast-Targeted Therapy**

*Principal experimental systems and translational limitations*

This table summarizes the principal mechanistic models discussed in Sections 3-4. Experimental systems are not treated as directly interchangeable. Reference numbers correspond to the revised manuscript. Directional findings are reported at the endpoint actually measured and should not be generalized to whole-skeleton remodeling or to a uniform human T2DM osteoclast phenotype.

| **Manuscript ref.** | **Model / cell source** | **Metabolic or experimental context** | **Endpoint measured** | **Principal observation used in review** | **Key translational limitation** |
| --- | --- | --- | --- | --- | --- |
| 25 | Osteoclast precursor culture during early RANKL stimulation | Early differentiation; increased glucose metabolism during osteoclastogenesis | Metabolic activity during differentiation | Osteoclast differentiation is metabolically dynamic. | Describes precursor metabolic demand rather than a diabetic disease state. |
| 26 | RAW264.7 osteoclast-lineage cell model | High-glucose exposure during RANKL-induced differentiation | Differentiation; ATP6V0D2/DC-STAMP fusion-related molecules | High glucose suppressed differentiation and fusion-related molecules. | Immortalized murine cell line; glucose exposure does not reproduce integrated human T2DM physiology. |
| 27 | Primary C57BL/6 bone-marrow monocytes | 33.6 mmol/L glucose with or without 1 μmol/L insulin | Osteoclast formation and pit resorption | Reduced osteoclast formation and pit resorption. | Defined supraphysiologic culture conditions; murine primary cells; isolated glucose/insulin exposure. |
| 28 | Rat T2DM alveolar bone-defect model with matched ex vivo assays | T2DM metabolic context during bone regeneration | Differentiation; resorptive function; matrix degradation | Reduced differentiation, resorptive function and matrix degradation during repair. | Alveolar defect/repair model; not steady-state axial/appendicular remodeling; species and site specific. |
| 29 | Streptozotocin-associated insulin-deficient rat model | 25 mmol/L glucose / STZ-induced hyperglycemia; insulin-deficient context | Osteoclastogenesis and bone resorption; CaMKII signaling | Increased osteoclastogenesis and resorption. | Model is framed as insulin-deficient/type 1 diabetes physiology; not direct evidence of typical T2DM. |
| 30 | db/db mouse cultures plus human peripheral-blood-derived osteoclasts in periodontal/inflammatory context | T2DM context with superimposed inflammatory/LPS stimulation | Baseline differentiation/resorptive capacity and inflammatory responsiveness | Human T2DM-derived cells showed similar baseline differentiation/resorption to controls but altered inflammatory responses. | Specialized periodontal/inflammatory environment; does not establish generalized focal osteoclast activation elsewhere. |
| 31 | RAGE-deficient mice and murine bone-marrow macrophages | Genetic RAGE loss of function | Maturation; cytoskeletal organization; resorptive function | RAGE participates in osteoclast maturation and function. | Genetic loss-of-function model, not a diabetic model; indicates pathway biology rather than T2DM directionality. |
| 32 | Mature osteoclasts on glycated bone/ivory matrices; precursor systems exposed to AGE-modified proteins | Nonenzymatic matrix glycation / AGE-modified protein exposure | Matrix degradation and osteoclastogenesis | Glycated mineralized matrix reduced degradation; AGE-modified proteins could inhibit osteoclastogenesis. | In vitro AGE preparation, concentration and substrate differ from human diabetic bone; in vivo contribution to circulating CTX is unquantified. |
| 33 | RAW264.7-derived osteoclast-like cells | AGE exposure at different differentiation stages | Fusion/differentiation versus post-maturation resorption | Stage-dependent effect: inhibition during fusion with modest enhancement of resorption when exposure occurred after maturation. | Murine cell line; effect direction depends on timing/stage; cannot define a single in vivo T2DM phenotype. |
| 34 | Human CD14-positive monocytes from healthy donors | Glycolaldehyde-derived AGE-BSA exposure | Osteoclast differentiation; IL-10-associated response | Suppressed differentiation. | Human cells but not derived from patients with T2DM; exogenous AGE preparation may not reproduce in vivo matrix exposure. |
| 35 | Marrow-derived macrophage lineage experiments | AGE exposure | Osteoclast- and osteoblast-lineage differentiation/function | AGEs suppressed differentiation/function across remodeling lineages. | Preclinical isolated exposure; supports network effects but not the net human T2DM remodeling state. |
| 36 | Nrf2-deficient mouse osteoclast precursors | Genetic redox perturbation / increased ROS | Differentiation; actin-ring formation; resorption | Enhanced differentiation, actin-ring formation and resorption. | Genetic redox model is not a T2DM model; cannot establish the net effect of chronic diabetic oxidative stress. |
| 37 | RANKL-stimulated osteoclast precursor system | Insulin exposure during differentiation | ERK1/2, NFATc1, ATP6V0D2 signaling and osteoclastogenesis | Insulin enhanced RANKL-induced differentiation in precursor systems. | Stage- and model-dependent insulin effect; does not represent hyperinsulinemic insulin resistance or β-cell failure. |
| 38 | Primary/cultured murine or rat osteoclast-like cells | Insulin exposure after osteoclast differentiation | Pit formation / mature resorptive function | Insulin-mediated reduction in pit formation reported in mature osteoclast-like cells. | Species/model dependence; mature-cell response cannot be assumed to match precursor differentiation effects. |
| 39 | Osteoblast insulin-signaling model | Insulin signaling in osteoblast lineage | Osteoclast-osteoblast coupling / systemic energy-bone interaction | Osteoblast insulin signaling influenced coupling and energy metabolism. | Indirect effect on osteoclast biology; not a direct T2DM-derived osteoclast assay. |
| 40 | Genetic adipose-lineage stromal-cell RANKL model | Stromal/adipose-lineage RANKL contribution | Osteoclastogenic support from marrow stromal niche | Adipose-lineage stromal cells can provide RANKL. | Genetic model supports niche plausibility but does not establish increased marrow RANKL as a universal human T2DM mechanism. |
